# From practice to lifestyle: conceptualizations of yoga in regular Ashtanga yoga practitioners using reflexive thematic analysis

**DOI:** 10.3389/fpsyg.2025.1582275

**Published:** 2025-06-04

**Authors:** Daniela Ramirez-Duran, Margaret L. Kern, Helen Stokes

**Affiliations:** ^1^Centre for Wellbeing Science, The University of Melbourne, Parkville, VIC, Australia; ^2^Melbourne Medical School, The University of Melbourne, Parkville, VIC, Australia; ^3^Faculty of Education, The University of Melbourne, Parkville, VIC, Australia

**Keywords:** yoga, Ashtanga yoga, regular yoga practitioners, reflexive thematic analysis, yoga philosophy

## Abstract

Yoga is usually defined and researched as a mind-body practice used for stress reduction, fitness and general health and wellbeing. While there are widely accepted definitions found in traditional texts and contemporary literature, as a millennial tradition yoga has evolved throughout time, places and people. Less is known about how regular yoga practitioners perceive, experience, and define yoga today. This study focused on a group of regular Ashtanga yoga practitioners (*n* = 200) to explore their conceptualizations of yoga in general, yoga philosophy, and their yoga practice using Reflexive Thematic Analysis. Yoga was defined as a practice for health and wellbeing, a method for personal inquiry and growth, a way of living, and a spiritual quest. Yoga philosophy was defined as the collection of ancient teachings for understanding human functioning and consciousness, providing a foundation for yoga practice and living life. Yoga practice was defined as the formal daily yoga ritual, adaptable, ever-changing, permeable and permeating into daily life. Three common threads revealed the entanglement of different elements and the significance of yoga as (1) an embodied integrated practice, (2) an embodied path for self-knowledge, and (3) a path for spiritual development. This entanglement and shared meanings underscore the relevance of considering participants' perspectives of yoga in research and applied settings to challenge assumptions and the prevailing image of yoga as mainly a physical and stress-reduction activity.

## Introduction

Though there are some widely accepted definitions of yoga included in traditional texts, perceptions about modern ways of practicing yoga can lead to different interpretations. As a millennial tradition rooted in India, yoga has evolved throughout time and context. Instead of remaining fixed, it has been permeable, through the exposure of various cultures, religions, and social-historical-political circumstances. While traditional and scholarly definitions can be identified when taking all these elements into consideration, lay people's perceptions of yoga in our globalized and digitalized world have also been documented. However, less is known about how long-term yoga practitioners conceptualize yoga according to their lived experience of engaging with different dimensions of the practice. Giving voice to people with a dedicated yoga practice can provide yoga scholars, researchers and teachers new insights about what yoga means in the context of our current modern world, as well as challenge biases that are being communicated to a broader audience. Here, we focus on examining how regular Ashtanga Yoga (AY) practitioners conceptualize yoga.

In this article, we first explore traditional conceptualizations of yoga, how these may be perceived as fixed or ultimate truths, and how we can gain a broader understanding of yoga by looking at these conceptualizations as situated in evolving contexts. Next, we discuss the emergence of modern postural yoga and the evolution of teachings over the last century to understand the main elements that have been highlighted and embraced by relevant teachers into yoga traditions that are being practiced today. We also briefly address the phenomenon of the science of yoga and yoga researchers/practitioners. Focusing specifically on a group of regular AY practitioners, we investigate their conceptualizations of yoga in general, yoga philosophy, and yoga practice using Reflexive Thematic Analysis. We discuss the main themes for each aspect of yoga, and then focus on the overarching themes across these three aspects of yoga. We end with a consideration of implications and applications of the research findings.

### From traditional to modern conceptualizations of yoga

Yoga is a Sanskrit word that has a wide range of translations, including “yoking,” “remedy,” “union,” “fitness,” and “meditation” (Monier-Williams, 1997). By using the term “traditional conceptualizations,” here we refer to definitions provided in the currently available traditional yoga literature. Before diving into how yoga is defined according to ancient texts, it is noteworthy that this review is not exhaustive, as yoga tradition is broad and extensive. Instead, our aim here is to provide a brief overview to understand that definitions contained in these texts are largely a product of different eras. According to Feuerstein (2003), yoga can be situated in a timeline containing different periods, including Vedic or Pre-Classical (3,000 BCE to 500 BCE), Classical (500 BCE to 800 CE), Post-Classical (800 CE to 1,500 CE) and Modern (1,500 CE to present). Each of these eras can be associated with particular worldviews and diverse contextual influences that shaped the way in which yoga was perceived (De Michelis, 2008; Jain, 2014a). Furthermore, it is important to consider that many early yoga texts including the wide-spread *Yoga Sutras of Patanjali* were transmitted orally and were written, interpreted, and translated at later times (Bryant, 2015; Devi, 2010). Thus, there is no universal definition of yoga.

Early traditional yoga definitions seem to mainly focus on describing the mechanisms of the mind and consciousness, specifically in terms of how the mind functions and how it can be stilled (Foxen and Kuberry, 2021). For instance, the *Katha Upanishad* (6.11) is a text from the Vedic era considering yoga to be “the firm restraint of the senses,” and the *Maitri Upanishad* (6.25) from the same period, states that yoga is “the unity of breath, mind and senses, and the abandonment of distracting thought” (Easwaran, 2007). Similarly, the Classical text *The Yoga Sutras of Patanjali* (1.2) defines yoga as “the cessation of the fluctuations of the mind” (Iyengar, 2005). The Post-Classical text *Yogabija* (89–90) estimates that yoga is “joining and therefore collapsing of all dualities until all that remains is oneness” (Mallinson and Singleton, 2016). It is believed that a range of philosophies influenced these ancient texts and the *Yoga Sutras* in particular, including Samkhya, Jain, Vedanta and Buddhism, all of which share the phenomenological inquiry about human suffering and its liberation through heightened levels of consciousness (Chapple, 2008; Vivekananda, 2012). According to Foxen and Kuberry (2021), these traditional definitions underscore the goal of reaching a particular state rather than detailing specific methods to reach such goal. The methods for practicing yoga have also been varied, and reflective of different religious, mystical and philosophical stances (Liberman, 2008).

Foxen and Kuberry (2021) provide a classification of yoga that synthesizes the meaning of yoga, the underpinning worldview and associated practices. The earliest stages of yoga can be understood as the “yoga of going.” Conceptualized as a spiritual practice, it was about transcending human experience and suffering in this world by going “upwards,” to a celestial plane. The evolution of yoga then led to the conceptualization of the human body as a scaled representation of the universe, which implied that the spiritual journey is not literal nor external. This is the “yoga of knowing” and represents an inner journey in which consciousness is transformed. This way of conceptualizing yoga can be associated with medieval traditions. Finally, the “yoga of doing” represents a collection of mind and body techniques primarily used by ascetics to tame the mind through the control of the body to escape the cycle of rebirth and attain liberation. Furthermore, these ideas of going, knowing and doing have been interwoven with other South Asian traditions and with other cultures as yoga kept evolving into the modern world (Foxen and Kuberry, 2021; Sarbacker, 2014; Singleton, 2010).

Hence, to understand modern yoga, it is relevant to consider not only the range of South Asian traditions in which it was conceived, but also the numerous formal and informal cultural exchanges between Eastern and Western traditions, worldviews and practices shaping the way in which yoga is perceived and practiced today as predominately postural (Brown and Leledaki, 2010; De Michelis, 2005; Jain, 2014b). Several scholars posit that modern yoga is the result of an historical period with particular elements interacting and reaching their maximum expression during the 20^th^ century (e.g., Singleton, 2010; Sjoman, 1999). *Hatha yoga* was being widely practiced during the Middle Ages and Early Modern era in India, placing its focus on the use of physical postures and breathing techniques (Liberman, 2008; Sarbacker, 2014). It has been argued that this yoga system deeply resonated with the physical culture movement that was emerging not only in India, but around the globe at the beginning of the 20^th^ century (Alter, 2004; Sarbacker, 2014). This way, modern postural yoga originated as “as a hybridized product of colonial India's dialogical encounter with the worldwide physical culture movement” (Singleton, 2010, p. 80). In other words, with the migration of yoga to the Western world in the late 19^th^ and during the 20^th^ centuries, yoga seemed to have circled from India to other parts of the world, to then return to India re-constructed, amplified and validated as an Indian tradition, transforming its original understanding and practice (Samuel, 2008; Sarbacker, 2014).

Thus, it seems evident that there is no fixed, intact, or single universal conceptualization of yoga (Maddox, 2015). Although some would argue otherwise (cf. Jain, 2014b), yoga has not been literally transmitted from a single tradition and regardless of the evolving social, cultural and historical contexts throughout centuries from its inception until today. Nowadays it seems that the yoga practice is the goal in and of itself, and that the physical practice in particular has become a way of “doing” as well as “knowing” an enhanced version of oneself (Foxen and Kuberry, 2021). Yet, the question around how yoga is conceptualized today remains.

### Defining yoga in a globalized world

Yoga has continued to evolve in a highly urbanized, globalized and digitalized world where capitalism and healthy lifestyles paradoxically co-exist (Burley, 2008; Jain, 2014a). As a product of the 20^th^ century, yoga is commonly defined as a mind-body practice or the combination of physical postures, breathing techniques, meditation and relaxation, which are mainly used for stress management, physical fitness, wellbeing and overall health (Alter, 2004; De Michelis, 2005; Jain, 2014a; Singleton, 2010). Yoga has also been advertised as an essential element of a healthy lifestyle, often being portrayed as being practiced by athletic slim young women with particular lifestyle choices (Hinz et al., 2021; Jeffrey, 2020), and thus making it more appealing particularly to highly educated middle-class women (Burley, 2008; Jain, 2014a; Park et al., 2019). Notably, the yoga industry seems to help in perpetuating these stereotypes. Miller (2016) defines the term “yoga industrial complex” coined by Broad (2012) as “the web of relationships between studio systems, yoga celebrities, certifying agencies, and large yoga businesses or industry including yoga product companies (…) as well as cultural producers” (p. 2). People engaging in professional activities within this complex contribute to the current representation of yoga, which can often marginalize and exclude certain types of yogis and make it accessible only to a particular group of people, namely white, young, and well-educated females (Miller, 2016).

Within the complexity of defining yoga due to its meaning being inherently bounded to social, cultural, and historical elements (Foxen and Kuberry, 2021; Jain, 2014b), contemporary scholars have generated a number of modern definitions. Probably, the definition of modern yoga scholar Jain (2014a) comprehensively articulates the breadth and entanglement of the elements of the practice as situated in the current world. She defines yoga as:

“*a collection of complex data made up of a congeries of figures, institutions, ideas, and practical paths involving mental or physical techniques – most commonly meditative, breathing, or postural exercises. It is believed to resolve the problem of suffering and to improve health, both defined in modern terms. Postural yoga often betrays a desire to repair what is perceived as an imbalance of ‘body-mind-soul.' Finally, it is tied to mythologies about the historical transmission of yogic knowledge, accumulating around a transnational community that has engaged in and transmitted what participants call yoga – and sometimes refuse to call yoga – since the twentieth century*.” (Jain, 2014a, p. 172)

Importantly, while integrating key aspects into a comprehensive definition, the question on “what participants call yoga” and how the philosophical underpinnings interact with their understandings and practice, remains unclear. While there is research looking at types of practitioners and motivations to practice (e.g., Cagas et al., 2022: Henrichsen-Schrembs and Versteeg, 2011), to our knowledge there are no studies investigating how regular yoga practitioners conceptualize yoga.

In the emergence and evolution of yoga, it is important to consider not only the philosophical context in which it sprouted, but also the relevant religious and scientific contexts. While the physical, mental, and spiritual were never considered to be siloed, the relevance of medical science as source of knowledge, with the subsequent emphasis on the physical body, has also contributed to the popularization of yoga as a mainly physical practice (Foxen and Kuberry, 2021). Notably, the “scholar-practitioner” phenomenon in Western academia has emerged alongside the evolution and globalization of yoga in the past couple of centuries (Singleton and Larios, 2020). This phenomenon is critical to acknowledge when understanding what scholars decide to spotlight as an object of inquiry in their research and how they conceptualize yoga. As Singleton and Larios (2020) contend, yoga scholar-practitioners are in a unique historical position where the knowledge about yoga emerges both from traditional sources and from empirical scholarship, and in which the academic and scientific circles tend to prevail as a valid form of advancing knowledge (cf. Atkinson, 2010, 2017; Jeffrey, 2020; Miller, 2016).

### Regular yoga practitioners and the Ashtanga yoga method

Historically, the typical representation of the ancient yoga practitioner was of a male ascetic secluded and devotedly living according to yogic principles and spiritual practices (Singleton, 2010). Although tantric yoga texts mention female practitioners embodying mystical powers, they were usually from low casts, and thus less accepted compared to renounced yogis (White, 2012). Similarly, householder yogis (namely those holding family, work, and social responsibilities) were perceived as less accepted due to socio-cultural-historical influences of their time (Jain, 2017). As yoga evolved, and only until fairly recently, the practice became more open and accessible to a wider range of people, including women and householders (Jain, 2017; Jeffrey, 2020). This can be largely attributed to yoga teacher Tiramulai Krishnamacharya, who relived and adapted yoga teachings following systematic methods while considering contextual elements of the modern world, and to his students who continued disseminating his teachings across the globe (Jain, 2017).

Krishnamacharya's experience and legacy embody the influence of contextual factors and evolution of yoga. He traveled extensively, with one remarkable journey to the Himalayan caves of Tibet to learn from his teacher Rama Mohana Brahmachari, a householder yogi (Desikashar et al., 2005; Jain, 2017). Krishnamacharya adhered to *parampara*, or a tradition in which teachings are passed on from *guru* (i.e., teacher) to *sisya* (i.e., student) with the expectation of the student showing discipline and respect toward their teacher (Jain, 2017; Singleton and Byrne, 2008). Renowned contemporary yoga teachers who have had great influence in modern yoga practice around the world learnt under his wing, including B.K.S. Iyengar, Indra Devi, T.K.V. Desikachar, and Pattabhi Jois. Many consider Krishnamacharya as the father of modern yoga, due to his key role in devising systematized movement-based practices with far reaching influences into our current knowledge and experience of yoga (Sarbacker, 2014; Singleton and Fraser, 2014). As Jain (2017) points out, the way in which these teachers lived their lives uncovered the possibility for contemporary practitioners to pursue a yogic lifestyle along with householder responsibilities.

Krishnamacharya taught yoga extensively under the support of the Maharaja of Mysore at a *yoga shala* (i.e., studio) in the palace, while also traveling across India disseminating the benefits of postural yoga with his students. From his perspective, the body was a place of inquiry and experience rather than being an object to be externally examined (Jain, 2017; Nevrin, 2008). These practices were passed onto Jois, who developed a particular method focused on specific elements of practice and teaching (Sarbacker, 2014). This method is known as *Ashtanga vinyasa yoga*, or simply Ashtanga yoga (AY), and it is characterized by sequences of asanas or postures that are taught progressively to students, usually within a group setting using a one-on-one approach. Students are encouraged to develop a daily self-practice at their own pace, following the principles of *tristhana*, which means three places in Sanskrit. This means performing *asanas* while directing attention to a particular *drishti* or gazing point, and with a continuous smooth flow of breath through the nose. Additionally, *asanas* are threaded with *vinyasa*, a system of moving and breathing in which each inhalation or exhalation is corresponded with one specific movement (Jois, 2016).

**Figure 1:**
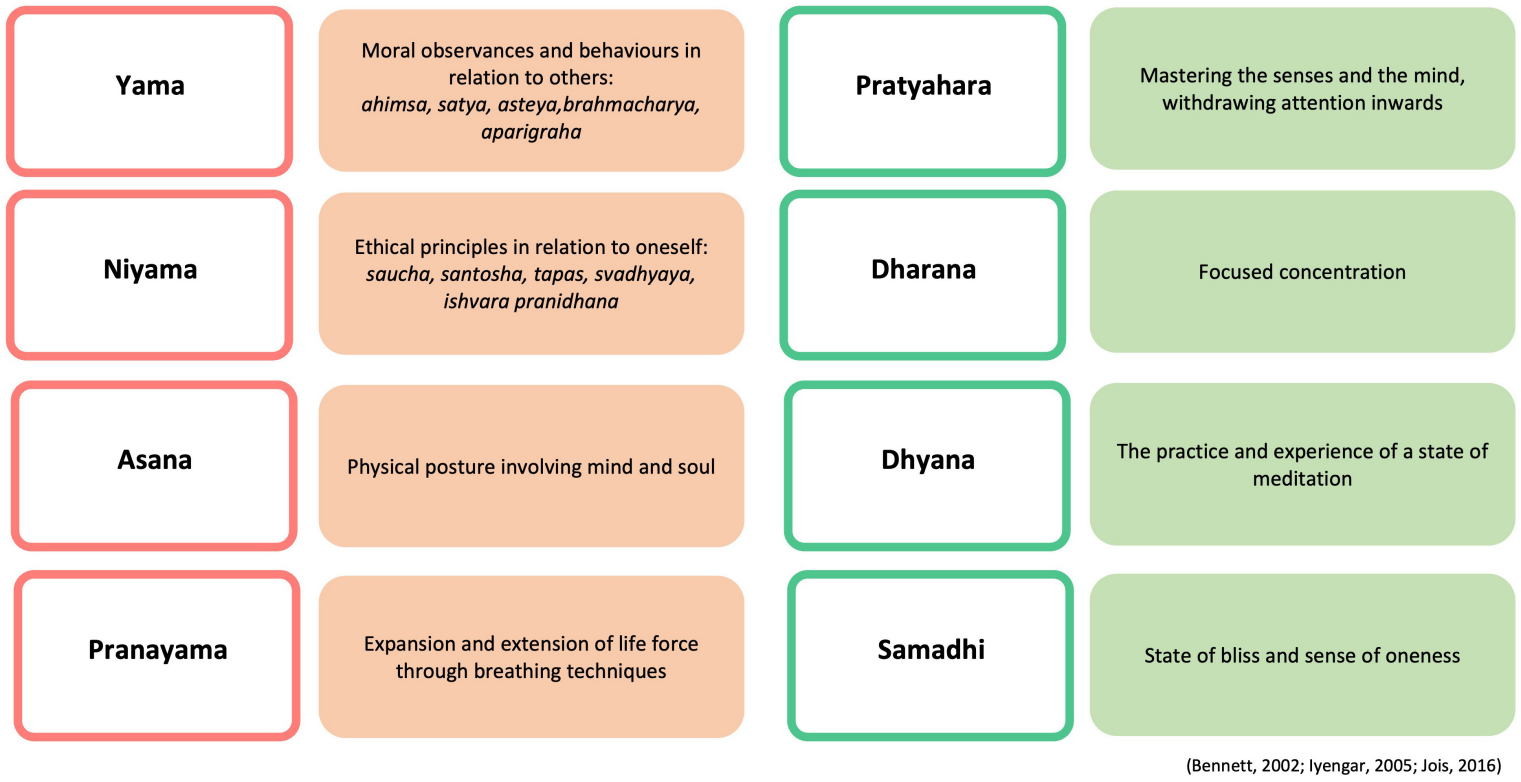
Ashtanga Yoga, the eightfold path of Patanjali. The word clouds were generated by NVivo12.

While the AY method is largely considered a system of physical discipline, Jois (2018) conceptualized yoga as *upaya*, or a path toward the realization of the true nature of oneself. Moreover, Jois (2018) and his grandson Jois (2016) stressed the importance of ethical principles and behaviors, as well as on meditative components, pointing out that without these elements, the yoga practice is merely a physical activity. In the AY tradition, the emphasis has been placed on the “inner practice of concentration and energy control,” framing the practice within Patanjali's eightfold path, also known as *Ashtanga Yoga* (Sarbacker, 2014, p. 104). Here, the physical body is seen as a gateway and a vehicle by which yoga is practiced and spirituality is experienced (Sarbacker, 2014), and where the eightfold path offers a guide to an integrated practice (see Figure 1).

As the meaning of yoga has varied throughout contexts and yoga practice is closely intertwined with traditions and philosophical frameworks, we aimed to investigate the conceptualizations of yoga in general, as well as of yoga philosophy and yoga practice in particular. We believe this adds breadth and depth, and allows the exploration of differences or similarities across these concepts. As conceptualizations of yoga according to regular practitioners have not been reported in previous research, our study seeks to explore these facets of yoga as experienced by practitioners and informed by academic literature. We believe that giving voice to practitioners' perspectives can expand the representation of yoga within and outside academia, providing insightful views. Since yoga practice and teaching methods are varied, here we intentionally focus on one particular style of yoga (AY), as its distinct method is systematic and can offer consistency across practitioners.

### The current study

This study follows up on previous studies investigating regular AY practitioners' (AYPs) perceptions of wellbeing (Ramirez-Duran et al., 2022a) and the dimensions of wellbeing (Ramirez-Duran et al., 2022b). Here, we examine AYPs' conceptualizations of yoga in general, yoga philosophy and yoga practice. Along with the previous studies, this study is part of a larger project examining the connections between yoga and wellbeing in regular AYPs, according to their own perceptions and experiences (Ramirez-Duran, 2023). The current study specifically focuses on examining regular AYPs' conceptualizations about these aspects of yoga separately, how these are interconnected, and how these perceptions can contribute to contemporary understandings of yoga.

To our knowledge, there are no studies about the conceptualizations of yoga from the perspective of regular practitioners. Our research inquiry was guided by constructivist and phenomenological worldviews, which highlight participants' lived experience and the co-creation of meaning through research. The study utilized an ideographic approach by focusing on participants' unique views on yoga, considering their AY background. Whilst this research uses a qualitative design, it integrated quantitative and qualitative elements during data collection and analysis. At the time of the research, the lead author (DRD) was a graduate researcher with background in psychology and wellbeing science, a qualified yoga teacher and a regular AYP for 8 years. This position was considered a strength in the research process, enabling a shared language and co-creation of meaning through developing the survey and collecting and interpreting data. The second author (MLK) has a background in psychology and wellbeing science, and the third author (HS) has a background in education and qualitative research and is a yoga practitioner.

## Materials and methods

### Participants

This study used a purposeful sample from a larger project focusing on AY and wellbeing, with 352 participants fluent in English and/or Spanish across 42 countries. Data were collected between April and August 2020 through an online survey (see Supplementary material 1). From the 352 participants who completed the online survey, 235 participants declared themselves as regular AYPs. A subset of 200 participants were included in this study, who met the criteria of being a regular AYP and completed at least one of three open-ended questions about yoga (see Supplementary material 1). From this sample, 188 participants described the meaning of yoga philosophy, 187 described the meaning of yoga, and 174 described their yoga practice. All participants were recruited online through social media platforms (i.e., Facebook, Instagram, Reddit, and LinkedIn) and electronic communication from yoga studios and/or teachers. Participants comprised a diversity of demographic and AY characteristics. All procedures were approved by the University of Melbourne's Human Research Ethics Committee (protocol #1955377.1).

The majority of practitioners identified themselves as female (79.5%), Caucasian (57.5%), between 25 and 44 years old (59.5%), declared living in the Americas (32.5%) or Europe (26%), held a higher education degree (85.5%), and did not follow nor identify with any specific spiritual tradition (41%). Most participants had been practicing AY regularly for 1 to 10 years (68%), between 3 to 6 days a week (78.5%) for 1 to 2 h each session (80.5%). The majority of practitioners followed an only Mysore format (44.5%) or combined it with traditional Sanskrit led classes (24.50%), and their practice mostly involved full primary series up to intermediate series (62%). The vast majority of practitioners stated that their practice included *vinyasa* (96%), *drishti* (93.5%), free breathing with sound (91%), the use of *bandhas* (90.5%), and chanting of opening and closing *mantras* (86.5%).

The majority of this sample of regular practitioners considered yoga philosophy relevant (74.5%) and engaged with it at least once a month (79.5%) by reading books and texts (81%), undertaking online courses (54.5%), following social media (54%), reading blogs (53.3%), and watching videos (49%). Over 70% of practitioners estimated to practice the four external limbs of yoga (i.e., *yama, niyama, asana, pranayama*) to a high extent (i.e., seven or above on a 11-point Likert scale). Finally, most participants perceived that their AY practice positively contributed to their wellbeing (i.e., seven or above in a 11-point Likert scale), especially to their physical (69.5%), emotional (68.5%), and psychological (65.5%) wellbeing (see Supplementary materials 2–6 for details).

### Measures

The online survey encompassed qualitative and quantitative items and was designed to assess an array of elements from yoga, wellbeing, health, personal traits, and demographic information. The survey was offered in both English and Spanish, with participants able to choose which language to complete the survey in. The current study incorporated questions about participants' AY practice, demographic information, and three open-ended questions exploring their perceptions of yoga in general, yoga philosophy, and their yoga practice (see Supplementary material 1). Details of the full survey have been described elsewhere (see Ramirez-Duran, 2023).

### Data analysis

Participants logged their responses using Qualtrics survey software (https://www.qualtrics.com/au/), which were then exported to NVivo (version 12) software for analysis. Data collection and analysis were conducted by the lead author, with sense-checking and input from the other two authors. Responses to the three open-ended questions were analyzed using Reflexive Thematic Analysis (RTA; Braun and Clarke, 2012, 2021). Word frequencies were examined and used to generate word clouds and summary tables (see Supplementary materials 7–9) to familiarize with the data as the first step of RTA. This allowed capturing conceptualizations in participants' own words for each aspect of yoga, thus providing a general understanding of data before diving into a deeper qualitative analysis (Eichstaedt et al., 2021; McNaught and Lam, 2010), whilst also offering a broad perspective for readers to better understand the reflexive and interpretative process.

**Figure 2:**
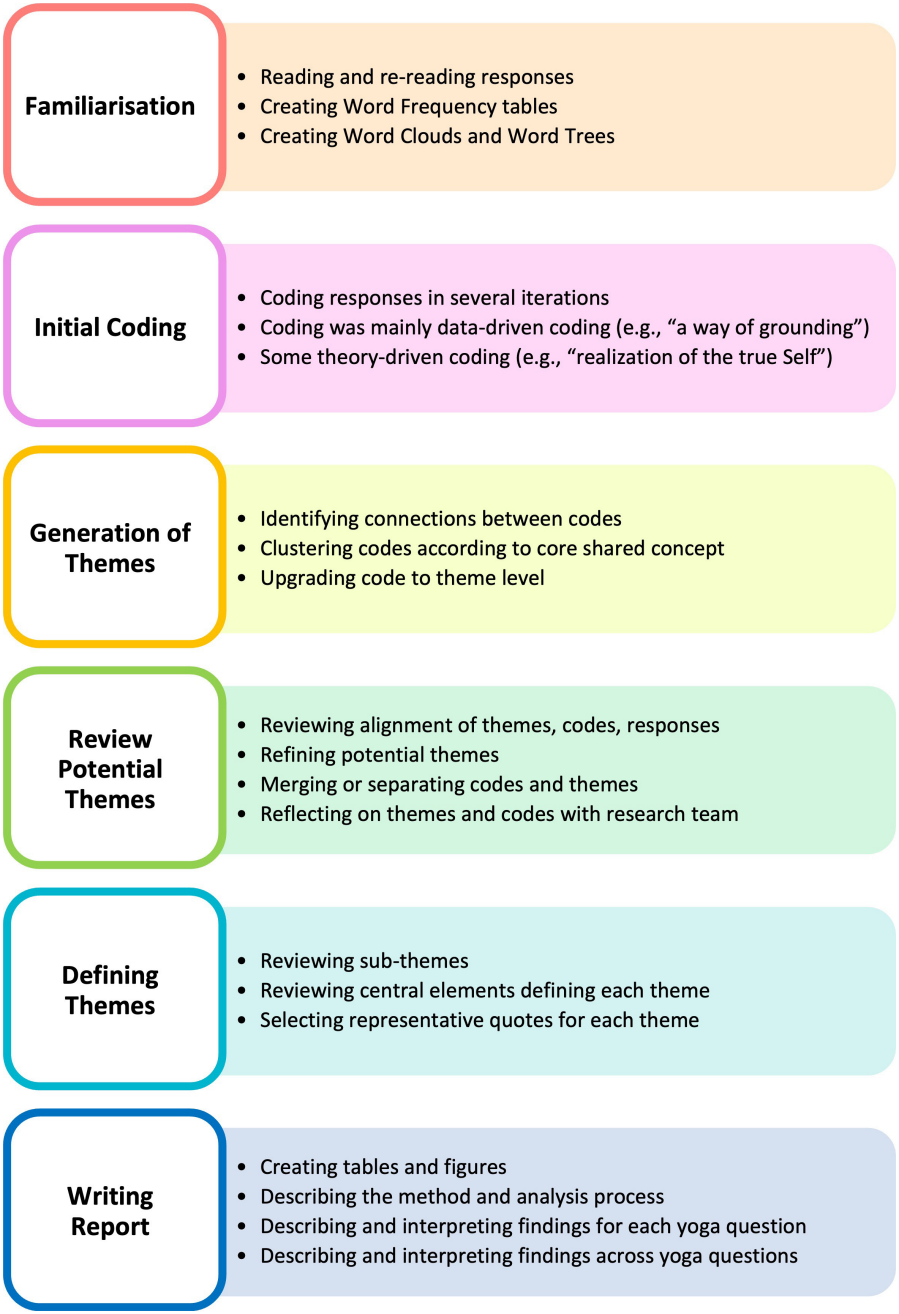
Application of the six steps of RTA in this study. The word clouds were generated by NVivo12.

RTA (Braun and Clarke, 2012, 2021) was used to systematically identify, reflect on, and interpret meaningful patterns across participants. We followed Braun and Clarke's (2012, 2021) guidelines, and applied six iterative steps to analyze data, which comprised (1) familiarization with the data, (2) generation of codes, (3) construction of candidate themes, (4) review of candidate themes, (5) definition and naming final themes, and (6) writing a report. More details about these steps are provided in the next section (also see Figure 2). This six-step process was undertaken for each aspect of yoga separately (i.e., yoga, yoga philosophy, and yoga practice), aiming to create core themes for each one of them. We then mapped themes across these aspects of yoga to visualize and identify any shared patterns of meaning and offer a general interpretation of the conceptualization of yoga according to this sample of regular AYPs.

### The process of generating and defining codes and themes

For the creation of codes and themes, the relevance of the content shared by and across participants was core. Following RTA, the data analysis was undertaken by the lead author, due to her expertise with psychology, yoga, wellbeing, and AY in particular. RTA acknowledges the researchers' role in shedding light, shaping and informing the data analysis from their own values, background, and interests, while spotlighting participants' voices (Braun and Clarke, 2021; Terry and Hayfield, 2020). Reflexivity is a central part of this type of thematic analysis, and in this case was achieved during the whole analysis process both by the lead author on her own and in collaboration with the other authors to sense-check thought processes and find alternate interpretations of the data (Braun and Clarke, 2021; Byrne, 2021). We acknowledge that the creation of the survey and the interpretation of results were influenced by our backgrounds, which enabled us to employ a common language with AYPs and to identify essential elements provided by participants.

**Figure 3:**
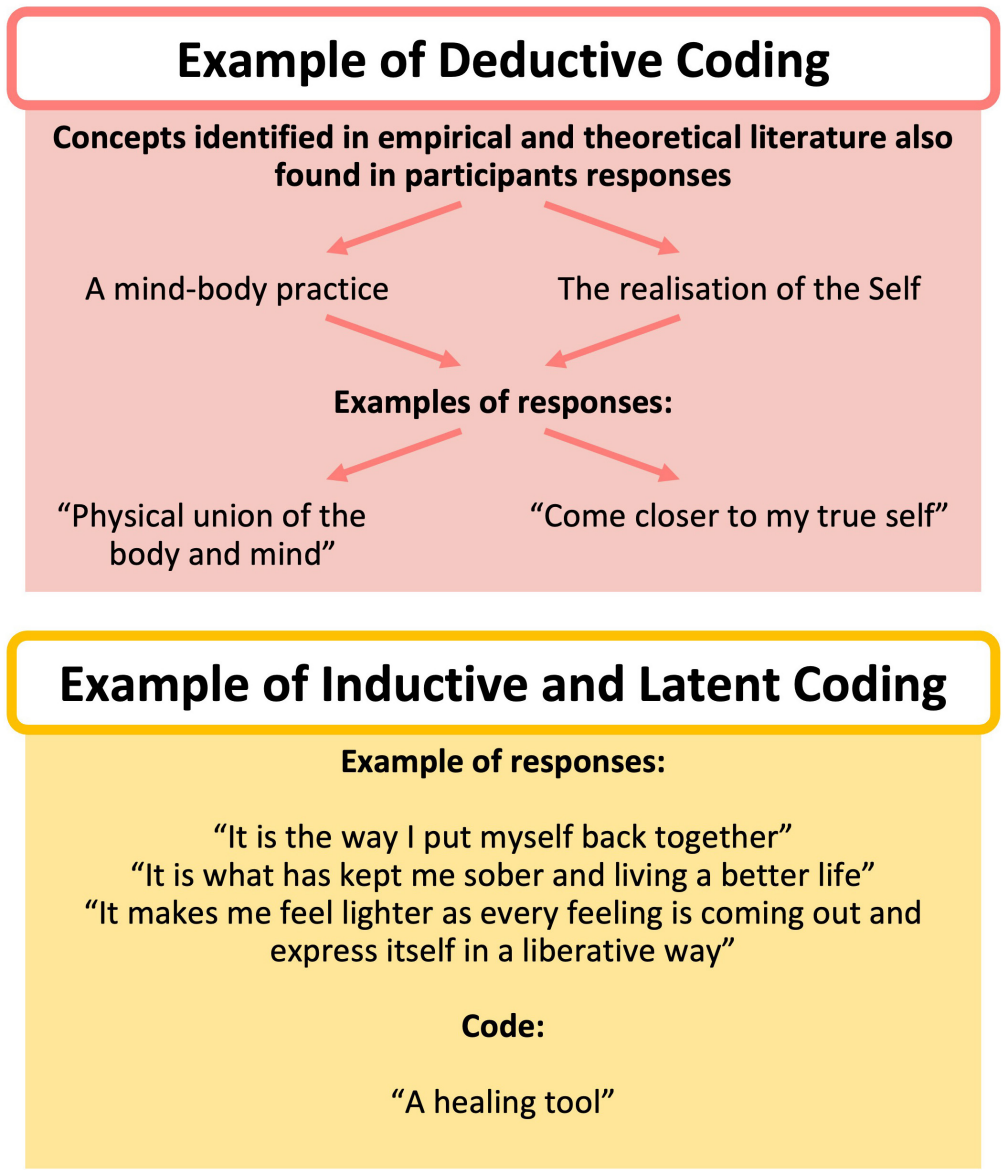
Examples of the RTA coding process. The word clouds were generated by NVivo12.

The initial coding stage started after familiarizing with data, as detailed above. Preliminary codes were created by utilizing both an inductive and deductive approach, and blending latent with semantic coding, a technique often used in RTA (Braun and Clarke, 2021; Byrne, 2021). The predominant approach for coding was data-driven (i.e., inductive) to best reflect participants' viewpoints. While theoretically-driven coding (i.e., deductive) was used by considering yoga literature and frameworks and consistent with RTA, codes were not pre-defined for data analysis (Braun and Clarke, 2021). For instance, for the analysis of the conceptualization of yoga in general, the codes “A mind-body practice” and “The realization of the true Self” represented a deductive approach, while the codes “A healing tool” and “A system of self-care and self-discipline” reflected an inductive approach (see Figure 3). The combination of latent coding with semantic coding was achieved through active interpretation of underlying meanings shared across participants while considering the significance of literal meaning in their choice of words. For example, while the code “A way of grounding, centering, and calming” captured descriptions of participants' views at an explicit level, the code “A healing tool” often entailed interpretation beyond a semantic level.

**Figure 4:**
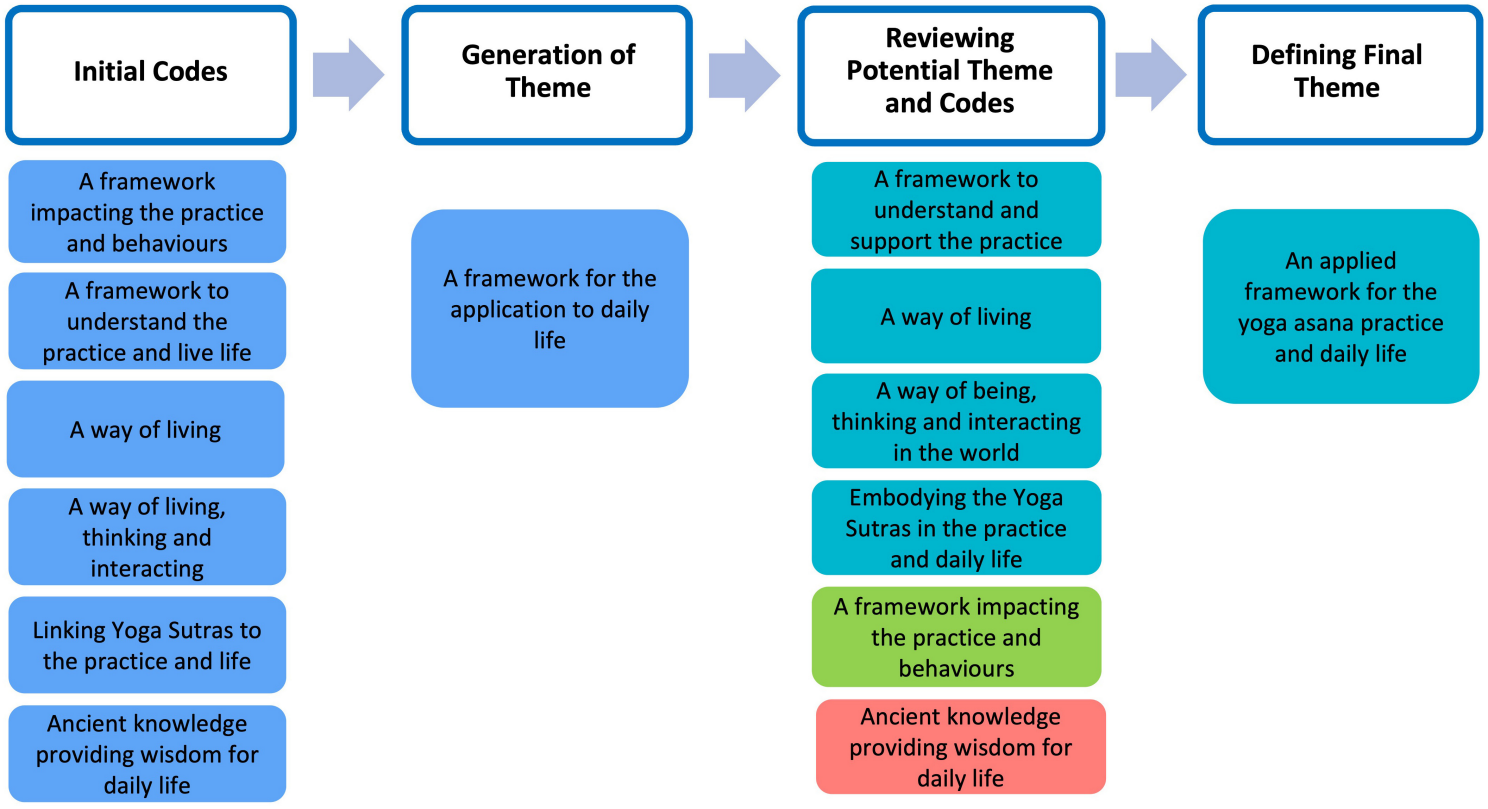
Example of RTA stages for creating, reviewing and defining codes and themes. The word clouds were generated by NVivo12.

After coding was finalized for each aspect of yoga, potential themes were created and revised along with subsequent iterations of coding to refine both codes and themes to best represent the meaning conveyed by participants. Then, final themes were defined and included in this written piece, which represents the final stage of the RTA process. Figure 4 illustrates this process using a theme from yoga philosophy. During the initial coding stage, we created codes to represent participants' voices and then grouped six of these codes under the potential theme “A framework for the application to daily life” to represent different ways in which the philosophical framework of yoga was applied or was influencing participants.

We then continued refining the codes and reviewing the theme, repeating this process with other codes and themes, aiming to capture participants' views more accurately while comprehensively communicating their shared meanings through coherent themes. For example, the preliminary code “A way of living” was created from semantic coding and separate from the preliminary code “A way of living, thinking and interacting” that involved latent coding. Upon close inspection, we changed the name of the latter to avoid confusion and best represent the meaning of the code by naming it “A way of being, thinking and interacting in the world.” The code “A framework that impacts the practice and behaviors” was merged with the aforementioned code and with the code “A framework to understand and support the practice.” The code “Ancient knowledge providing wisdom for life” was moved to another theme as we deemed that it best captured the shared meaning of yoga philosophy as a framework to understand the origins and background of the practice. Finally, when refinement and review of final codes and themes was completed, we defined each theme, selected representative quotes (see Table 1), and developed a summary table including word frequencies for transparency.

**Table 1:** Conceptualizations of yoga, yoga philosophy and yoga practice with themes and quotes from regular AYPs (^*^).

**WB dimensions**	**Themes**	**Representative quotes**	**N**	% (^**^)
Yoga	*Yoga is a holistic and multidimensional practice*	“A method for improving the physical part of myself and eventually influence the spiritual part” “To me yoga means that I can explore the relationship between my body and mind” “It is a door into looking at your life from a different place, to expand the perspective about existence and our human abilities, it is an opening into love in all its shapes and manifestations”	116	10.55%
	*Yoga is a tool for healing, coping and cultivating health and wellbeing*	“Before I practiced Ashtanga I had physical pain walking and so it helped me alleviate that” “A tool to feel better, to cope with life, a belief, a lifestyle” “Yoga is a way I balance both my mental and physical health, and has become the dominant method I use for both”	54	5.91%
	*Yoga is a method for knowing, accepting and developing the self*	“Getting to know myself and break free from my own limitations” “For me yoga is the sum of tools, practices, perspectives, inner and external states that facilitate and drive self-knowledge, liberation of imposed and pre-established patterns” “For me it is a path for self-knowledge and personal realization”	74	6.73%
	*Yoga is a way of being, living and seeing the world*	“It's a way of life and I find is very grounding and calming” “A philosophical system that provides a framework for navigating life as a human” “It's my lifestyle, it's the way I connect to myself and to others” “To be in tune with yourself and with the world”	108	9.82%
	*Yoga is the journey toward the realization of the Self*	“Freedom from the suffering caused by my mind” “Calming the mind and the union with everything” “I see yoga as a collection of meditative practices designed to unite the conscious experiencer with their truest nature”	76	6.91%
Yoga philosophy	*A collection of teachings for the knowledge and evolution of the self*	“An ongoing journey of bettering life and self” “Yoga philosophy means learning to know and love thyself. Trust and be kind. Shape, learn and evolve” “Self-discovery and acceptance” “Peeling back the layers to uncover the truth of reality and Self”	43	3.91%
	*An applied framework for the yoga asana practice and daily life*	“Yoga philosophy is the knowledge that has been gathered over thousands of years that explains the system of yoga and puts some reasoning behind why we do what we do” “I guess it provides a way of being in the world and guidance around how to behave and consider oneself and others” “Trying to reconcile the ideas put forth in the Yoga Sutras with what we wxperience in our personal practice and how we live our lives”	112	10.18%
	*A spiritual path toward the understanding of reality, human nature and beyond*	“Yoga philosophy for me means getting in touch with my true nature and being reflective on where I am in my spiritual journey” “An inner work/journey while learning to trust forces beyond my existence” “Yamas and niyamas are universal (…) I see the same themes in various traditions/religions and that ultimately they represent what most people seek in their lives in order to live a rich and full life (...) it just so happens that for me, the tradition of yoga is my path to enrich my life”	39	3.55%
	*A framework to understand the foundations and origins of the practice*	“The tradition and the spiritual aspects of my practice” “The history and theory yoga is based on” “The root and core of the practice. Without yoga philosophy my practice would feel more like stretching or exercise without any deeper aspirations.”	46	4.18%
	*Attitudes and engagement toward Yoga Philosophy*	“It is not very important” “It still seems really complex to me, but I always try to learn more” “The study of the Yoga Sutras” “That's a loaded question. I'm not sure how to answer. It means a lot to me. I study yoga philosophy to complement my practice.”	31	2.82%
Yoga practice	*Description of the elements included in the yoga practice*	“I do my full primary practice almost 6 days a week right after waking up in the morning” “Mostly Mysore for asana 5 or 6 days a week. Pranayama practice at same frequency. Mindful meditation 4 days a week” “My practice starts with 20 min of meditation, followed by 90 min of asana, and finishes with 30 min of pranayama”	260	23.64%
	*Outcomes and applications of the yoga practice*	“Yoga threads through everything. Thoughts, words, actions, re-actions, state of consciousness, understanding life, health of the body and the mind” “I do my best to be introspective and ethical in action” “It always makes me feel good. Despite the mental and physical discomfort, everything keeps healing.”	37	3.36%
	*Adapting the practice and learning from challenges*	“I struggle with some physical inabilities/injuries, so I have to practice very carefully. I'm not looking for getting new poses also because of I see how strong the simplest practice can be” “Will perform the sun salutations and finishing postures as a bare minimum and if I'm pushed for time”	69	6.27%
	*Expanding the yoga practice through additional elements*	“I have lost flexibility over the years. I added a yin yoga practice several times a week, and that seems to be helping a great deal.” “Daily chanting of Bhagavad Gita and Patanjali's Yoga Sutras. Reading of Vedas, Upanishads, Puranas, Epics” “During the day I try to carry the philosophy of yoga with me, in my actions, expressing gratitude, feeling the connection and compassion to everything, observing myself in my actions and choosing how to react.”	35	3.18%

^*^See Supplementary material 13 for details of sub-themes. ^**^Percentages were calculated based on the total mentions (N) within each of the three aspects of yoga.

## Results

We first provide a descriptive analysis of the words used by participants to describe yoga, yoga philosophy, and yoga practice, and then turn our focus onto the reflexive analysis of themes for each of these aspects of yoga. This exploration of words and their frequency was considered a key element in the familiarization phase of RTA, complementing the typical practice of reading responses in multiple occasions, with the aim of becoming closely interconnected to the data (Byrne, 2021). As stated in our previous studies (Ramirez-Duran et al., 2022a,b), we contend that the exploration of word frequencies offers a landscape of the dataset when using large datasets and when responses vary in length, and provides the reader a clear illustration of findings to follow the description, interpretation and discussion of each theme.

**Figure 5:**
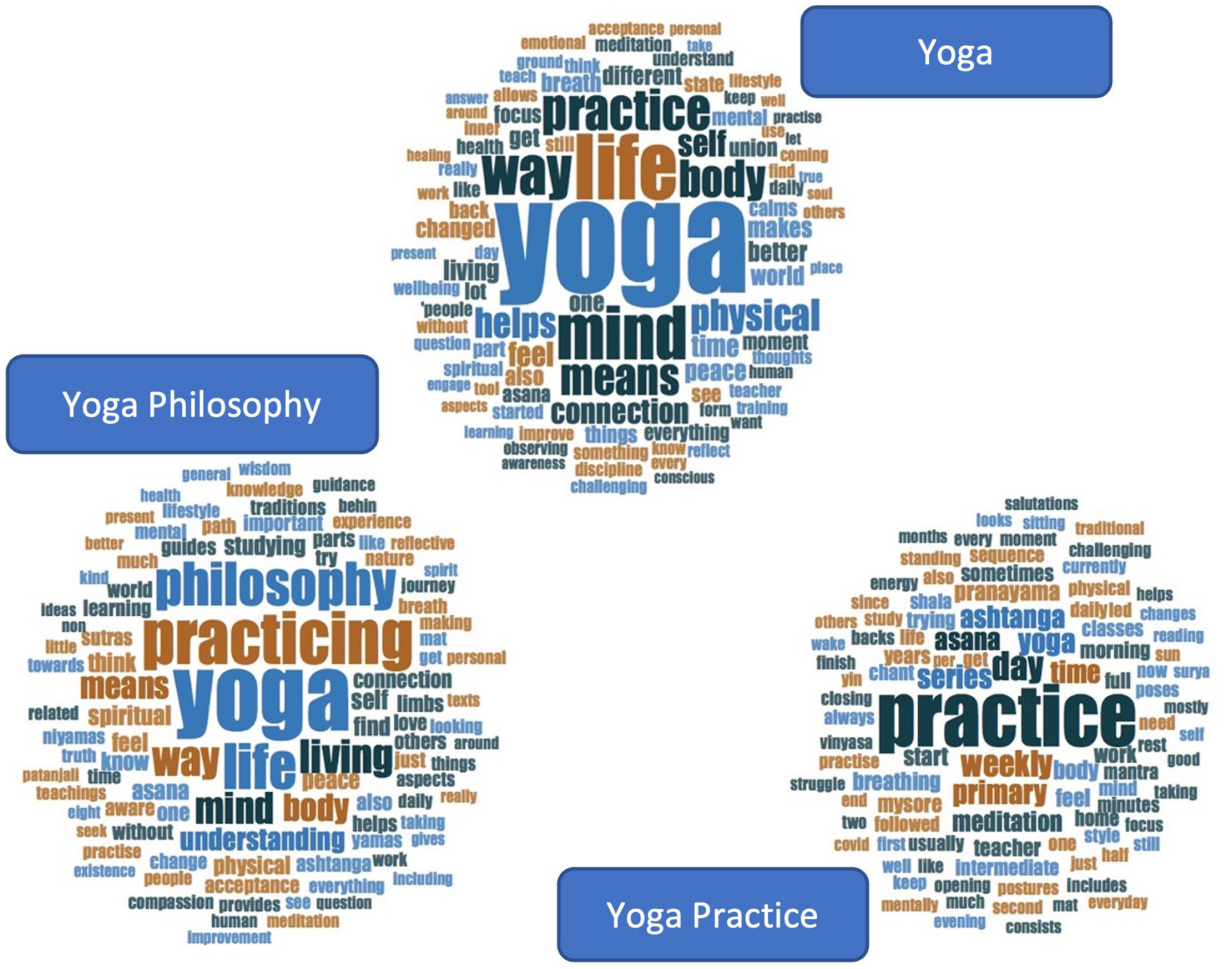
Word clouds of the conceptualization of yoga, yoga philosophy and yoga practice. The word clouds were generated by NVivo12.

Participants' responses considerably varied in length (min = 1, max = 1,032), with an average of 30 words to describe each element of yoga (see Table 2). The word clouds in Figure 5 comprise the 100 most frequently used words by yoga practitioners to define yoga, yoga philosophy, and their yoga practice. Words using a larger font represent a higher frequency (min = 4, max = 225), with no specific meaning for color. Some word clouds contain more words with a larger font size, which is explained by the variation of word frequency and distribution of words (see Supplementary materials 7–9 for details).

**Table 2:** Overview of regular AYPs' responses word counts for each aspect of yoga.

	**Yoga**	**Yoga philosophy**	**Yoga practice**
N	187	188	174
Min.	1	1	1
Max.	262	1,032	297
Mean	24.25	27.78	38.02
Median	14	13	26

We identified clusters of similar words within each aspect of yoga, as well as similarities across the three aspects that we investigated. For example, words such as “life” and “living,” “physical” and “body,” “mind” and “mental,” in addition to “self,” “peace,” and “connection” were commonly employed across yoga in general and yoga philosophy. The yoga practice aspect was commonly depicted by the use of words reflecting the asana sequence and additional practices, such as “day,” “weekly” and “time,” “primary,” “intermediate,” and “series,” and “asana,” “meditation,” “pranayama,” and “breathing” (see Supplementary materials 7–9 for details). Across the three aspects of yoga, it was also common to find the word “feel,” which was mainly used to describe an emotion, a sensation or an experience (see Supplementary materials 10–12 for word trees).

### Conceptualizations of yoga in general

The first theme was “*Yoga is a holistic and multidimensional practice”* and was defined as representing a multifaceted practice entailing physical, mental, and spiritual components, which can be experienced on their own or as intertwined with others. For example, for some participants, yoga was only experienced as a physical exercise, noting for example that yoga was “*a form or exercise that I enjoy, calling it anything more than that would be problematic*.” Other participants specifically identified yoga as a form of mind-body practice by referring to it as “*a form of exercise, not only physical, but also psychological and spiritual*.” For other practitioners, yoga represented purely a spiritual practice, for instance writing that “*yoga is divine connection, discipline and evolution*.” Others focused on yoga as a contemplative practice, perceiving yoga as “*moving meditation*.” Or in words of another practitioner, yoga “*provides a constant platform from which I can observe how my body and mind are feeling in that particular moment; it serves to remind me each time I practice to be more humble and forgiving of myself and to keep things in perspective*.” This theme reflects the nature of yoga as offering different avenues for practitioners to engage with, and addressing physical, mental and spiritual dimensions separately and as a whole.

The second theme was “*Yoga is a tool for healing, coping and cultivating health and wellbeing*” which defines yoga as a tool that practitioners use for healing at a physical and/or a mental level, for coping with challenges and navigating life, and for fostering, improving and balancing health and wellbeing. Some participants specifically referred to healing effects. For example, one participant spoke about yoga as “*a way of healing ourselves*,” while another asserted that “*yoga has provided a physical avenue to processing complex childhood trauma*.” Other participants emphasized the coping skills and resilience that yoga can provide. For instance, one practitioner said, “*I strongly believe that my yoga practice makes me a better person and more equipped to deal with the challenges that life throws at us*.” Participants also perceived yoga as a health and wellbeing tool. One practitioner spoke about yoga as “*a way to maintain my mental health*,” while another said that “*yoga is a way to nourish my mind and body, I have dealt with anxiety and depression for years, but the method of breathing and physical activity of yoga have helped me cope*.” This theme represents the relevance of yoga as a set of skills that can assist practitioners not only to deal with and recover from health conditions and trauma, but also to foster and maintain health and wellbeing.

The third theme was “*Yoga is a method for knowing, accepting and developing the self* ” and was defined as a system that promotes self-care and self-discipline, that enables a process of self-inquiry and self-discovery, and can lead toward the development and transformation of oneself. For instance, the concept of discipline and care toward oneself is reflected in the word of one participant who stated, “*yoga means commitment, to myself and the practice, showing up every day and constantly finding ways to improve my practice and myself.”* Other participants highlighted aspects of reflection, inquiry and understanding of oneself. For example, one participant spoke about “*the equilibrium and the self-knowledge that allows me to connect with my own being; transformation and growth,”* while another described it as “*a way of knowing my body and its limitations, as well as of my mind, it is a constant learning about my being and acceptance of life.”* In terms of development and transformation of the self, some participants pointed out more egotistic elements, while others identified more transcendental elements. For example, the former was captured by a participant who expressed that “*yoga makes me feel proud of myself, every time that I improve on my asanas,”* and the latter is reflected in a practitioner who defined yoga as “*a tool for self-knowledge and that gives me the possibility to observe myself and motivates me to want to evolve as a human being.”* This theme seems to encapsulate one main purpose of yoga from different perspectives, which is related to the understanding and the evolution of the self.

The fourth theme was “*Yoga is a way of being, living and seeing the world*” and it was defined as the internal lens to perceive and connect with oneself, the principles and guidelines by which one lives life, and the external lens to perceive and connect to others and with the world as an extension of oneself. One aspect of this theme was the perception of yoga as a way of being more grounded, centered, and calmed. For example, one practitioner spoke about yoga as “*a way for me to be better to others, and in return, I feel more grounded and able to deal with stress,”* and another said that it “*helps to ground me and gives a lot of meaning to my life.”* Another essential aspect of this theme was the perception of yoga as a way of living. In words of one practitioner, “*it means a way of life that helps me deal with all that is happening in the world and how to appreciate everything we have, even our negative feelings.”* Another practitioner described it as “*a way of life, it means to practice awareness and to be fully present in my life.”* Some participants also highlighted the aspect of connecting with others and having a sense of community. For instance, one practitioner noted “*it is a way of living, which rules the way you relate with others,”* while another spoke about yoga as “*a wonderful anchor that helps me stay grounded, centered and mindful; it provides me with a sense of wellbeing, curiosity, community and safety.”* This theme depicts yoga as transcending the practice level and positioning itself as an embedded framework that can influence practitioners' perceptions and experiences of themselves, of others, and the world, and guide their behaviors.

The fifth theme was “*Yoga is the journey toward the realization of the true Self* ” and was defined as looking beyond the nature of the human mind by gaining stillness and clarity to see oneself and the true nature of the Self. Some practitioners highlighted yoga as “*a way to calm the mind”* or as “*stilling the mind and living in equanimity.”* Stilling the mind and seeing clearly entails the acknowledgment of the nature of the human mind before recognizing and tapping into the true nature of the Self. This ulterior form of awareness was expressed through two ideas. One was the experience and practice of a state of union or oneness. In words of one practitioner:

“*[yoga] is the union of the body, mind and spirit. It is a method for calming the mind, for observing myself and get to know my own essence, for connecting with my soul and with what unites me with everything in this physical plane and in a universal plane, which transcends the physical.”*

The other core idea was the realization of the Self, which was portrayed by one practitioner as “*the state of consciousness, union, oneness with the Self,”* and by another as “*discovering the true nature of oneself.”* This theme conceptualizes yoga as a path or a process in which the practitioner is able to observe and embrace the nature of the human mind, developing higher levels of awareness that lead to the realization of the connection of the individual consciousness (i.e., self) with the universal consciousness (i.e., Self).

Thus, yoga was conceptualized as a practice that practitioners do, as a tool that practitioners use to improve health and wellbeing, as a method for personal growth, as a lifestyle and worldview, and as a spiritual journey. These forms of conceptualizing yoga are consistent with empirical and scientific literature where practitioners have been found to engage with yoga in one or more dimensions (Ivtzan and Jegatheeswaran, 2015) and the use of yoga as therapy (Bennetts, 2022; Van der Kolk, 2017) and for wellbeing (Gaiswinkler and Unterrainer, 2016; Ivtzan and Papantoniou, 2014). Conceptualizing yoga as a practice including physical, mental, spiritual, and contemplative elements as well as encompassing particular worldviews and behaviors encapsulates the evolution of yoga into a hybrid modern practice (Singleton, 2010). It also aligns with the yoga literature conceptualizing yoga as a practice fostering self-knowledge and transformation (Vivekananda, 2012), cultivation of higher levels of awareness and realization of the Self (Kidd and Eatough, 2017).

### Conceptualizations of yoga philosophy

The first identified theme was “*A collection of teachings for the knowledge and evolution of the self* ” and depicted yoga philosophy as a set of teachings involving the cultivation of self-awareness and observance, and providing an avenue for the reflection, discovery, understanding and evolution of oneself. Some participants highlighted the contemplative teachings. For instance, one participant spoke about “*the self-understanding of life experience and the process of thoughts in the mind.”* Other participants perceived yoga philosophy as a way to connect with, accept, and understand oneself, while for others it also meant a path toward self-evolution and transformation. The following description provided by a practitioner encapsulates all these elements:

“*It is a very beautiful and loving way of looking at human existence, the processes of evolution and growth. It helps me to understand my deepest feelings and to accept the temporary transit called life, fostering self-love and it has helped me to find a sacred place, time and space for myself. Without a doubt the practice opened internal portals that nowadays make me feel more conscious and in synch with how my soul and humanity feel*.”

This theme suggests that yoga philosophy can allow a deeper understanding of practitioners, as well as promote personal growth and transformation when they engage with its teachings.

The second theme was “*An applied framework for the yoga asana practice and daily life*,” defining yoga philosophy as applied knowledge that enables a better understanding of the yoga practice and permeates into practitioners lives by influencing their lifestyles and ways of being, thinking and interacting with the world. For some practitioners, yoga philosophy was considered a fundamental component for supporting and deepening the practice. For instance, one practitioner stated that “*yoga philosophy adds another level to the Ashtanga method, it is essential to deepen the understanding of the practice.”* Other practitioners stressed the role that principles and values play in their lives. For example, one participant referred to yoga philosophy as:

“*the mindset/values by which I practice, and the intention to carry that mindset/values off my mat. The more I practice yoga, the more those values are engrained in who I am. I learn what I need to focus on or change as I practice. These lessons on my mat reveal truths about my life off the mat.”*

A subset of practitioners specifically mentioned elements from the Yoga Sutras in their conceptualizations, for example:

“*For me, the Ashtanga method provides a clear path for applications of the yamas and niyamas in everyday life. I find that the asana practice prescribed by Ashtanga provides experience of non-violence, truthfulness, non-stealing, moderation, forgiveness and compassion (ahimsa, staya, asteya, mitahara, ksama, and daya)*.”

This theme represents the embodiment of principles and teachings contained in the philosophical sphere of yoga both during the daily practice and in daily life, as well as the dynamic interconnection between philosophy and practice.

The third theme framed yoga philosophy as “*A spiritual path toward the understanding of reality, human nature and beyond*,” representing existential and spiritual reflections and connections within oneself, with others, and a divine plane. Some participants underscored the premise of stilling the mind and embracing impermanence as essential to understand reality. For example, one practitioner said that “*yoga philosophy is a structured and well researched method to create clarity within the mind and enable true insight into the nature of reality.”* Other practitioners referred to the interconnection between body, mind and spirit as one core principle of yoga philosophy. One participant spoke about “*the combination of breath and movement to unite the body, mind and spirit; the deepening of a spiritual flexibility and spiritual awakening so that one can navigate through life with grace, integrity and peace.”* Some participants specifically addressed the issue of realizing the true nature of oneself, as being one with a universal consciousness. In words of one practitioner: “*Yoga is a roadmap to liberation. It is the path to self-knowledge, to the re-encounter with our essence and recognize ourselves with divinity. For awakening from our spiritual amnesia and remind us of our natural state: love.”* This theme represents the spiritual quest in which participants may embark through the engagement with yoga philosophy, whether that represents developing a deeper connection to oneself and achieving mental steadiness and clarity, or with everything surrounding oneself, including the divine.

The fourth theme was “*A framework to understand the foundations and origins of the practice*” and was defined as perceiving yoga philosophy as an ancient cosmovision that needs to be acknowledged and provides a foundation for the contemporary practice. For one practitioner, yoga philosophy “*is the understanding where yoga came from, what a yoga practice is all about and what benefits can provide the mind and the body.”* Another participant noted that “*I like knowing as much about the philosophy underlying yoga as I can […] because I feel that taking the time to understand it is the most respectful way to engage with a culture and tradition that are not originally my own.”* Other participants also pointed out similarities with other philosophical or spiritual traditions that were relevant to them and connected with their understanding of yoga. For example, one practitioner stated that “*I can relate parts of yoga to Buddhism, and being with the breath is the same as trying to live in the moment, which you can find in Buddhism.”* Another aspect that was spotlighted by some participants was the way in which yogic worldviews are closely intertwined with yoga practices. In the words of one practitioner, yoga philosophy was depicted as it follows:

“*It's a way of life… and we cannot become sages overnight… so I personally believe that every day we work on ourselves. May it be on the mat or in situations or reacting to unwanted things… I find yogic philosophy in it, some way or the other it is related to everything we think, feel and do in life*.”

This theme reflects the importance of acknowledging the heritage and understanding the worldviews that gave birth to yoga as a comprehensive system of philosophy and practice, which may resonate in particular ways due to the practitioners' belief system and life experiences.

The fifth theme was “*Attitudes and engagement with Yoga Philosophy*” and represented the range of attitudes and behaviors held by participants in regard to yoga philosophy. A number of practitioners explicitly reported having minimal engagement with yoga philosophy or considering it as not relevant. For example, one practitioner said that “*I don't pay much attention to the philosophy,”* while another mentioned *that “I'm not particularly affiliated with any yoga philosophy, as I don't think about it often when I practice.”* Other practitioners expressed the feelings that it evoked, such as “*overwhelmed*,” “*inspirational*” or “*humbled*.” Participants also referred to the importance of self-study and to acquiring their own understanding of aspects of philosophy that could resonate with them and thus, apply to their lives. For instance, one participant noted that “*I started studying [yoga philosophy] because I started to see certain relationships between meditation, yoga and Buddhism,”* while another stated that “*I am rather not dogmatic […], my philosophy covers several fields and would not only refer to a single discipline.”* This theme represents the diversity of views and complexities in understanding and relating with yoga philosophy as a broad umbrella term gathering different teachings that can be connected to other fields.

In summary, yoga philosophy was conceptualized as a collection of ancient teachings reflecting ways of understanding human nature and higher levels of consciousness, organized into an applied framework that serves yoga asana practice, guides ways of living, and supports personal and spiritual development. Consistent with previous research on yoga practitioners, attitudes toward philosophy and immersion in yoga as a broader system are varied, which relates to different experiences regarding the practice and its benefits (Gaiswinkler and Unterrainer, 2016). Although some may argue that AY is mostly a physical practice (Sarbacker, 2014), it seems that regular practitioners value traditional teachings about spirituality, the nature of reality, and ethical principles, combining Eastern and Western ideas with their own personal values.

### Conceptualizations of yoga practice

For conceptualizations of yoga practice, the first identified theme was “*A description of the elements of the yoga practice*” and reflected the most representative set of features that are usually practiced. It mainly reflected descriptions of the asana sequence, the modality of the practice, the frequency and time dedication, as well as chanting, meditation and pranayama at the beginning and/or at the end of the practice. For example, one practitioner described their practice as “*I go to a shala about 3 to 4 times a week for Mysore style practice, plus led class once a week.”* Another practitioner said:

“*My asana practice consists of surya namaskar A and B, standing poses, full or half primary and second until dwipada, urva dhanurasana plus drop backs and closing […] In addition to my asana practice I do alternate nostril breathing, try living my life being kind toward myself and others, and try observing my mind and not grasping onto my emotions.”*

This theme seemed to reflect and expand on what participants had previously reported in the online survey by completing closed ended questions, occasionally providing further details that were not captured in the survey.

The second theme was “*Outcomes and applications of the yoga practice*” and reflected the benefits practitioners experience from the practice as well as the applications off the mat and into daily life. This was comprehensively expressed by one practitioner:

“*I practice on my own, in my lounge room, as I live in a regional area without an Ashtanga yoga teacher. I attend classes or workshops when I can, or if I'm visiting the city. I have young children, so my practice often occurs alongside someone asking where their socks are, what's for lunch or to tell me that their brother hit them! I consider this to be part of my practice. Nothing is perfect. The idea of always practicing in a perfect, quiet yoga shala rarely occurs for me! If I want to practice yoga, I need to choose to accept that my environment is not always the stereotypically perfect yoga environment. But it's perfect to me.”*

Another practitioner spoke about the practice in terms of the benefits, saying “*I'm less concerned about the physical aspect, although it has been incredible, and more about what the practice brings me mentally and spiritually.”* This theme reflects how the practice is lived by practitioners beyond the formal asana practice, how it is carried out in real life settings and the benefits that they perceive that they are gaining from having that regular practice.

The third theme was “*Adapting the Ashtanga Yoga practice and learning from challenges*,” which was defined as tailoring the yoga practice to the practitioner's personal needs and circumstances and learning from the challenges that may lead to such adaptations. While practitioners described how they would modify their practice, they still recognized following the foundations of the AY method. For some practitioners, these adaptations were directly related to the COVID-19 pandemic, in terms of shifting to a home practice, online practice, or due to mental challenges related to lockdowns. For example, one practitioner spoke about “*After COVID my practice has been a home practice, I struggle without having the environment of teacher and other students to inspire me to stay present for the entire practice.”* Other participants described the way in which they navigated changes and adapted the practice accordingly, due to injuries, motherhood, time, work commitments, or other constrains. In words of one practitioner: “*Now that I'm in my late 50*′*s, it is more challenging with aches and pains, but I can feel how good it is for me. I'm steady but the energy levels shift a lot. I just show up and do what I can.”* Other practitioners specifically referred to how they were challenged by the practice or how they struggled while practicing. For example, one practitioner mentioned “*It is quite challenging physically and mentally. Some postures are very demanding, and I work a lot to get better in them.”* This theme represented how practitioners at different times in their life may encounter obstacles to practice the way in which they were once able to do so, representing an opportunity for growth and learning, with many persevering through and adapting to the challenge.

The fourth theme was “*Expanding the Ashtanga yoga practice through additional elements*” and was defined as complementing the AY method with other practices that help to bring a balance or enhance the experience of the practitioner. For example, some practitioners referred to incorporate another asana practice alongside the AY practice. One practitioner mentioned that “*two to three times a week I will practice Yin/restorative yoga in the evenings as I find this helps to balance out the Yang nature of the Ashtanga Yoga practice.”* Other participants stated that studying yogic texts and yoga philosophy was part of their practice. For instance, one participant said *that “I read philosophy and writings on philosophy daily, I try to incorporate mindfulness into my daily life.”* Other practitioners mentioned the relevance of their teacher in supporting them to find ways to adapt or balance their practice. For example, one practitioner expressed that “*I have in the past modified my practice alongside the advice of my teacher to make it easier to manage in times of stress to remove the thinking element of whether I should show up or not.”* Similar to the prior theme, this theme speaks about building a personal practice by seeking elements, not only within, but also outside the AY method to support, deepen and expand the yoga practice.

Overall, the yoga practice was conceptualized not only in terms of the elements and features contained in the daily AY asana practice, but also in terms of how the practice is malleable and evolving according to particular circumstances and needs, how it can draw upon wisdom from other practices and how the practice permeates into daily life. Although most of the aspects contained in this general conceptualization of yoga practice have not been reported in studies, it seems to match anecdotal evidence on what happens in AY studios. It also seems to represent the constant evolution of the yoga practice through various contextual influences (Jain, 2014a). Importantly, the applied aspect of the practice onto daily life is aligned with the argument that the AY practice was developed and intended to be practiced by people with social and family commitments (Maehle, 2006).

## Discussion

Findings from our RTA yielded between four and five themes for each of the three aspects of yoga that we focused on for this study. In this section, we synthesize and integrate the themes identified in yoga, yoga philosophy and yoga practice by looking across them in more detail. We discuss shared perspectives amongst participants, to extend our current understanding of yoga from regular AY practitioners' viewpoints.

### Shared meanings across yoga, yoga philosophy and yoga practice

**Figure 6:**
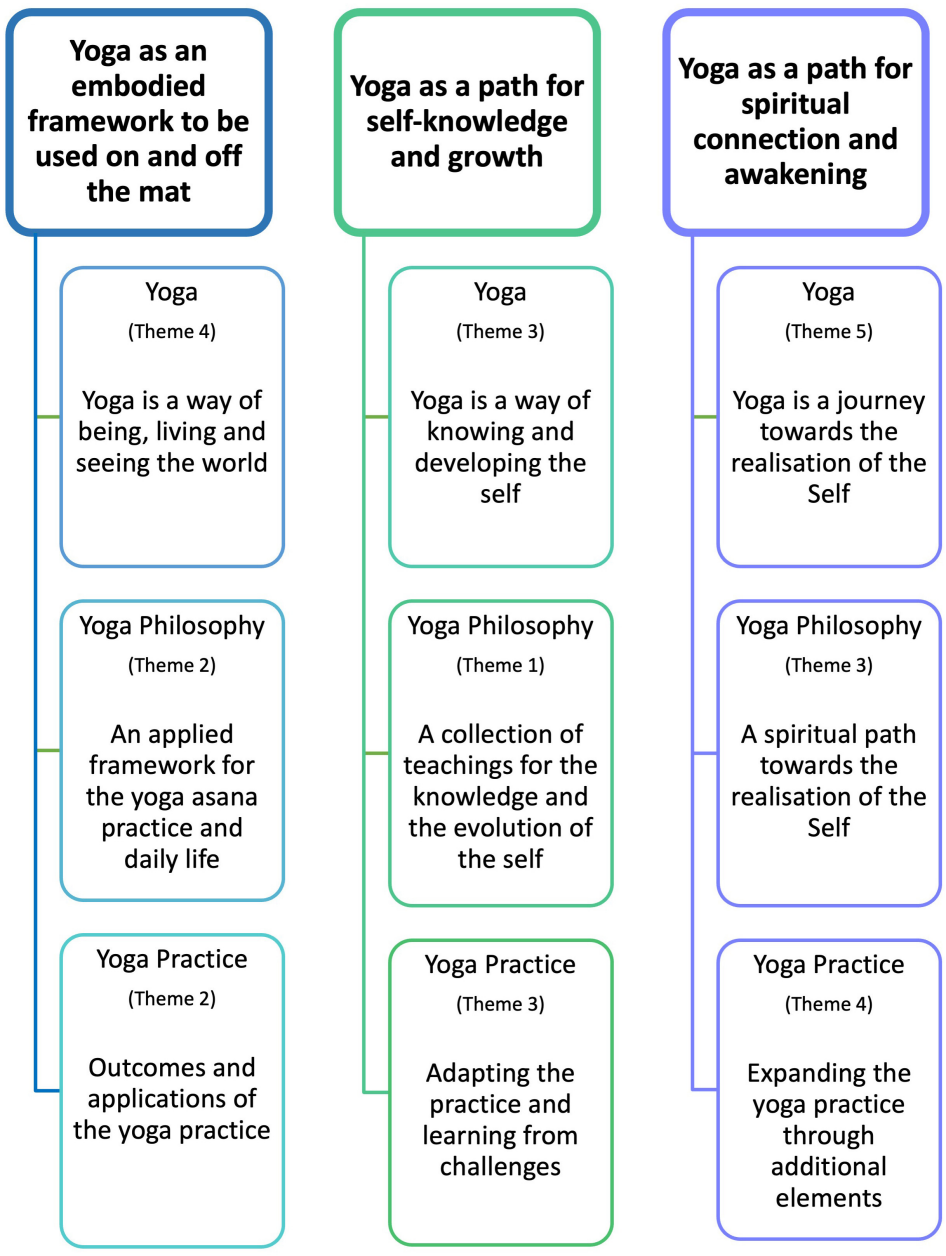
Overarching themes across yoga, yoga philosophy and yoga practice. The word clouds were generated by NVivo12.

After conceptualizing each aspect of yoga according to practitioners' perspectives, we examined themes across yoga, yoga philosophy, and yoga practice. We identified three common threads depicting yoga as intertwining theoretical and philosophical aspects with traditional and contextual practices that practitioners adopt according to their own lived experiences and belief systems. We have grouped these common threads into three overarching themes, which are discussed in the next sections and illustrated in Figure 6 (also see Supplementary materials 14–16 for examples).

### Yoga as an embodied framework to be used on and off the mat

The first common thread across the three yoga aspects was found amongst the themes “*Yoga is a way of being, living and seeing the world*” from yoga in general (fourth theme), “*An applied framework for the yoga asana practice and daily life*” from yoga philosophy (second theme), and “*Outcomes and applications of the yoga practice*” from yoga practice (second theme). We synthesized this common thread into the overarching theme “*Yoga as an embodied framework to be used on and off the mat*,” which conceptualizes yoga as a worldview that imbues not only the formal practice, but also the way in which practitioners lead and live their lives. By embodied, here we refer to both the embodied practice that usually takes place on the mat in the form of *asana* practice combined with other elements (e.g., *pranayama*, meditation), and to the embodiment of values, principles and beliefs connected to the yoga tradition (e.g., *yama, niyama*) through the enactment of habits and behaviors in real life and beyond the formal practice. Within this overarching theme, participants often referred to yoga and yoga philosophy as “*a way of life*,” and their descriptions provided details of how this was enacted through the application of the practice to thoughts, emotions and behaviors, or experienced via mental and spiritual benefits. Aligned with contemporary yoga literature, yoga is conceptualized beyond the physical practice, as a lifestyle system sustained in philosophical and ethical guidelines emanating from diverse sources of teachings (Feuerstein and Wilber, 1998; Jain, 2014b; Jeffrey, 2020).

What practitioners eloquently expressed in terms of lifestyle and ethical practice is consistent with both traditional and contemporary literature. For instance, Jeffrey (2020) argues that yoga has been referred to as a way of living as early as in pre-classical and classical yoga texts, such as the *Rig Veda* (1,500–1,000 BCE) and the *Yoga Sutras of Patanjali* (200–400 BCE), evolving ever since into contemporary embodiments of yoga practices and worldviews by regular practitioners. In this study, the most cited text was the *Yoga Sutras*, with some practitioners referring to the *Vedas* or other traditional texts as ethical guidelines for a way of living. This might be due to the widespread of the *Yoga Sutras of Patanjali* in the West through different gurus, such as Swami Vivekananda (White, 2014). Besides practitioners referring to what can arguably represent some of the eight limbs of yoga when describing the yoga practice (i.e., *asana, pranayama, pratyahara, dharana*, and *dhyana*), they mentioned qualities that explicitly or implicitly represented aspects of *yama* and *niyama*. These principles that apply to the attitudes and behaviors toward others and to oneself and have been encouraged to be followed within and outside the AY tradition (Iyengar, 2005; Jois, 2016). Similar to our research, Jeffrey (2020) also noted how long-term yoga practitioners adopted and adapted ancient and traditional ethical teachings into their own lives and contexts in their own meaningful ways. These yogic ethical adoptions and adaptations offer the opportunity to reconsider what is the purpose of yoga in our current day and age, and the influence that yoga practitioners' viewpoint can bring to the table.

This overarching theme also indicates that this group of regular AY practitioners have what Buckingham (2017) refer to as an “integrated practice,” or the postural practice underpinned by the ethical practice. As discussed in the previous sections, this lifestyle can take manifold shapes according to participants' perspectives, including aspects concerning health and wellbeing, personal environment, interactions with others, patterns of thoughts and behaviors, and the relationship to higher consciousness (Vivekananda, 2012). Adaptations to the applications of traditional yoga teachings with the evolution of modern yoga as experienced by practitioners has its counterpart in the scholarly world. For instance, the interpretations of the *Yoga Sutras*, and thus the 8-fold path of Patanjali across time and place, have multiple views that can vary significantly depending on the author and their context (Jeffrey, 2020). Additionally, some scholars interpret both *yama* and *niyama* as principles that can be applied simultaneously to oneself and others (e.g., Bennetts, 2022). For example, *ahimsa* traditionally speaks about no harm, yet recent literature speaks about embodying positive qualities such as kindness, empathy, and compassion, not only in regard to others, but also toward oneself (Desikachar, 1995; Bennetts, 2022). Thus, how yoga practitioners interpret and use these teachings can influence contemporary conceptualizations of yoga as much as what yoga scholars and teachers interpret and communicate about the theory and the practice.

### Yoga as an embodied path for self-knowledge

The second common thread was identified between the themes “*Yoga is a way of knowing and developing the self* ” from yoga in general (theme 3), “*A collection of teachings for the knowledge and the evolution of the self* ” from yoga philosophy (theme 1), and “*Adapting the practice and learning from challenges*” from yoga practice (theme 3). We integrated these themes into the overarching theme “*Yoga as an embodied path for self-knowledge*,” which conceptualizes yoga as a system comprising both a framework with teachings and insights into human nature and its evolution, and the set of practices that enable the experience and application of such framework in the formal yoga practice and in daily life. The core idea of this overarching theme is the embodied understanding and development of oneself. This means that self-knowledge occurs not only as cognitive and meta-cognitive processes by reflecting upon and observing oneself, but also occurs at an experiential level by feeling body sensations, abilities and limitations, and by enacting self-caring lifestyle behaviors and mindful interactions with others. This further implies that self-knowledge and self-transformation can take place both during the formal practice and the lifestyle practice.

While this overarching theme illustrates what has been documented in modern yoga literature as representing yoga as “a system of personal inquiry and experience” (Vivekananda, 2012, p. 11), it also resonates with Krishnamacharya's and Jois' view of the physical body as a place of attention, observation, and experimentation (Sarbacker, 2014). Here, however, practitioners have highlighted that this personal inquiry and experience transcends the physical practice, and flows into daily life, either as an intentional practice or as a state. Fiori et al. (2017) propose that since the practice of AY is personal and often embedded as a lifestyle, the increased dedication and immersion may lead to an enhanced level of self-awareness, self-compassion, and a greater focus on self-realization. Although we cannot draw casual relationships in this study, this might be the case for this group of practitioners. In a previous study with the same group of regular practitioners, participants' embodied perceptions of wellbeing spoke about heightened levels of self-awareness through the body (Ramirez-Duran et al., 2022a). Their conceptualizations of yoga as an embodied path for self-knowledge also speaks about this continuum between increased awareness of external and internal body sensations, emotions, thought patterns and behaviors, leading to increased consciousness of how oneself operates in and relates with the world. Thus, this path of self-knowledge may have ripple effects from within oneself, to social relationships, to the environment.

This further echoes Buckingham (2017)'s conceptualization of yoga as a “technology of the self” (p. 144), which is defined as “a microprocess, an ethical and aesthetic practice, a practice of the self […], a way of acting with faithfulness to oneself” (p. 143). Here, practitioners articulated how yoga in general terms, and its philosophical and practical elements, encompass methods to constantly calibrate and re-calibrate oneself through observation, acceptance, and actions. These actions can be seen as dynamic entangled processes involving what Barad (2007) calls intra-actions (i.e., in relation to oneself) and inter-actions (i.e., in relation to others). As argued by Buckingham (2017), the integrated yoga practice (i.e., on- and off-the-mat) seems to interweave the personal work practitioners do on themselves with their relationship with others and the world around them. By integrated, she refers to the personal, social and political practice, entailing an embodied, ethical, and ecological approach (Buckingham, 2017).

On the one hand and at a personal level, this common thread conceptualizes yoga as a path for developing an enhanced version of oneself, equipped with tools to navigate life. This speaks to recent research looking at postural yoga as contributing to self-regulation and resilience in regular practitioners (Sullivan et al., 2018; Tyagi et al., 2016; Upadhyay et al., 2022). On the other hand, the ethical framework of yoga (i.e., *yama* and *niyama*) may support practitioners into a constructive engagement with themselves, others, and their environment. *Satya*, one element of *yama*, is closely intertwined with this, as it speaks to truthfulness and integrity in the relationship with oneself, others, and every other aspect of life (Bennetts, 2022). Elements from *niyama* are also represented in this overarching theme. While references to self-acceptance can arguably represent *saucha*, which has been interpreted as honesty, forgiveness, and self-compassion; self-discipline can be seen as *tapas*, which has been interpreted as determination, consistency, regular practice and mindful decision making (Bennetts, 2022). Hence, self-knowledge arises from within and from the relationships we establish in our worlds.

### Yoga as a path for spiritual awakening and connection

The third common thread was found across the themes “*Yoga is a journey toward the realization of the Self* ” from yoga in general (theme 5), “*A spiritual path toward the understanding of reality, human nature and beyond*” from yoga philosophy (theme 3), and “*Expanding the yoga practice through additional elements*” from yoga practice (theme 4), in particular the sub-themes related to the study of texts and yoga philosophy and to the connection to other belief systems. This thread was synthesized into the overarching theme “*Yoga as a path for spiritual awakening and connection*,” which defines yoga as an avenue that practitioners may choose to further develop themselves in terms of spirituality and transcendence. This spiritual journey can take diverse shapes according to each practitioners' belief system, context and experiences. The central idea shared by participants here is the relevance of the practice and state of mental steadiness and clarity as a steppingstone into the experience of union and oneness within oneself, with others, and a divine plane, and into the awareness of the true nature of reality and being. This eventually encompasses the realization of the Self as the true nature of being and a broadened or heightened consciousness, which may be facilitated not only through the formal practice, but by the engagement with the spiritual and philosophical foundations.

Within this overarching theme we can see what Sarbacker (2014) refers to as spiritual development collapsing into physical development in different modern postural yoga traditions, including AY. Through this knowledge, the links to spiritual and physical foundations are made visible and coherent (meaning making) for practitioners. Here, participants' conceptualizations account their lived experiences through the practice with links to the meta-physical elements of yoga-related frameworks. While considering yoga as the evolution of the self, this spiritual-physical collapse can be interpreted in relation to the *pancha kosha* (i.e., five sheaths) framework conceived within Vedantic philosophy. This model encompasses five levels of awareness in human existence, from gross to subtle: the physical body, the vitality body, the mental body, the wisdom body, and the bliss body, leading to the realization of our spiritual nature at the core (Feuerstein, 2013; Vivekananda, 2012). Through the expansion of awareness through each of these layers, yoga practitioners would be able to embark in the quest toward the realization of the true self, pure awareness or Self, which in Vedantic philosophy is the *Atman*. Noteworthily, this concept is also acknowledged in other yoga-related frameworks such as *Samkhya* and *The Yoga Sutras of Patanjali*, as well as in forms of Buddhism and Jewish mysticism (Feuerstein, 2013), which might explain how this state of oneness and higher consciousness resonates with practitioners from diverse backgrounds and beliefs.

It has been argued that modern postural yoga has gained greater popularization due to meeting the expectations around physicality and remaining secular yet mystical and spiritual enough to be appealing for practitioners seeking personal and spiritual development (Sarbacker, 2014). This seems to be the case for this group of practitioners. Indeed, the philosophical and spiritual underpinnings of yoga combined with its physicality is what distinguishes it from other forms of purely physical or purely spiritual practices (Jeffrey, 2020). What practitioners reported in this study also speak about what the main proponents of modern postural yoga, and AY in particular, envisioned and underscored: the relevance of spiritual development through the physical practice (Sarbacker, 2014). While embodying these teachings, still some practitioners conceptualized yoga as an either purely physical or mental practice. This aligns with previous research showing that practitioners may start a regular practice for physical and/or mental health reasons, developing spiritual motivations as they deepen their practice (Büssing et al., 2012; Ivtzan and Jegatheeswaran, 2015; Park et al., 2014, 2019). It also aligns with research showing different types of practitioners, some focusing on physical elements and others on mystical components (Cagas et al., 2022; Henrichsen-Schrembs and Versteeg, 2011). This goes back to *hatha* yoga and the idea that the physical practice brings about mental and spiritual benefits, irrespectively of which is the underlying motivation for practicing (Sarbacker, 2014).

Finally, linking back this overarching theme to the ethical framework and practice of *niyama* in particular, key elements shared by practitioners reflect how these teachings permeate their experiences and perceptions of yoga. For example, *santosha* can be understood as contentment or equanimity, involving the acceptance of internal and external circumstances, and thus mental steadiness (Bennetts, 2022; Jois, 2016). *Svadhyaya* is interpreted as self-study leading to self-knowledge, which may entail personal habits, as well as thought and behavioral patterns, implying the ability to observe and accept oneself before being able to transform aspects of oneself (Bennetts, 2022; Jois, 2016). *Isvara pranidhana* means surrendering and devoting oneself to the divine or to a higher purpose, being able to transcend the ego, often implying a spiritual devotion, which from a psychological point of view can be considered as relevant for building resilience (Bennetts, 2022; Jois, 2016). These aspects seem to summarize what has been previously interpreted and articulated throughout this common overarching theme.

### Integration of common threads

These three common threads represent the interconnection or entanglement of philosophy, theory, and practice in yoga as experienced and perceived by this group of regular AY practitioners. These threads reveal that themes and aspects of yoga cannot be viewed entirely siloed and separate from one another. This entanglement truly represents yoga as a system of philosophies and practices that are in constant interaction with the context in which they continuously emerge, evolve, and become, as experienced, interpreted, and communicated by regular practitioners, scholars, teachers, and communities. In this group of regular practitioners, it seems that perceiving yoga as a firmly embedded lifestyle encompassing the formal AY practice (i.e., on-the-mat) as well as embodied adoptions and adaptations of traditional teachings, creates a fertile ground for an inner journey toward self-knowledge and spiritual growth. The “*practice of the self* ” seems to happen in the space between where the physical and the spiritual collapse. Through micro-processes or intra-actions, practitioners gain an increased understanding of themselves, allowing them to transform aspects within themselves and in their relationships with the outer world. This might lead to heightened levels of awareness and into spiritual journey toward the realization of their true self. Hence, similar to what has been reported in the mindfulness literature, yoga may be considered both a practice that can be performed and a state that practitioners can experience occasionally, as well as a way of being.

### Implications and future directions

This study contributes to existing literature by focusing on the experiences of long-term yoga practitioners to understand what yoga means in the modern world. While the open-ended questions enabled participants' voices to come through as rich insightful responses, the lead author's background provided depth and nuance to our findings. Next, we discuss a number of implications emanating from this research.

Conceptualizations of yoga by this group of regular AY practitioners reveal the entanglement of philosophical, ethical, and spiritual elements along with the formal postural practice, usually comprising *asana, pranayama*, and meditation. This suggests that scholars and researchers should not precipitate drawing assumptions about how practitioners perceive yoga as essentially a physical activity for physical fitness, health, or wellbeing. While this still might be true to many practitioners, it seems that higher levels of immersion with yoga (i.e., moving from practice to lifestyle) bring about an integrated practice that is experienced as a state of being-in-the-world. This aligns with previous research unveiling different types of practitioners (e.g., Henrichsen-Schrembs and Versteeg, 2011; Cagas et al., 2022). Thus, future research could focus on longitudinal investigations on yoga practitioners to further understand the perception, evolution and entanglement of these elements, as well as the experienced pathways in which yoga provides benefits.

Furthermore, as an ancient tradition that has interacted in various contexts and with diverse cultures, worldviews, and practices, yoga keeps evolving within the modern world. While practitioners and scholars invest their efforts in acknowledging and understanding its roots, it seems necessary to also embrace the process of continuous de-construction and construction of yoga philosophy and practice as experienced by practitioners and scholars today. This implies incorporating practitioners' and research participants' viewpoints on what yoga means to them, before imposing a pre-determined conceptualization devised unilaterally by researchers. These pre-determined definitions reflect assuming that yoga only entails a few elements, which might not be completely relevant to all practitioners. Similar to recent research on lay conceptualizations of wellbeing (e.g., Sollis et al., 2024), applying a participatory lens in yoga research can contribute to the design of more inclusive and accessible yoga programs. These can be co-created with and tailored to participants' needs, characteristics and contexts, whilst considering their understanding of yoga.

Another important implication is the dissemination of research to a broader audience. Learning about what do long-term practitioners perceive and experience in relation to yoga may help in debunking myths set by mass media and popular beliefs. This means moving away from yoga stereotypes in which only few people with certain attributes (e.g., healthy, fit, flexible, and young women) are improperly perceived as able to practice or to benefit from the practice. In doing so, there is an opportunity to broaden the scope and attract the attention of a wider range of practitioners. The “scholar-practitioner” may play a key role in shifting paradigms in terms of yoga research and practice, contributing in different ways of generating knowledge in the field and making it more accessible.

### Limitations

There are several limitations in this study that need to be acknowledged. Participants in this study were self-selected, which means that they were motivated to take part in the research and had access to digital platforms. This might have affected their responses as well as the variety in characteristics of the sample. Most of the participants included identified as middle-aged females, Caucasian, and well-educated. This position may reflect more privileged experiences in life and with yoga, which might have influenced the content of the responses in terms of how yoga is practiced and conceptualized. We recognize that, for future research, a greater variety of practitioners needs to be considered. Nonetheless, the issue of privilege in yoga confirms what has already been reported in existing literature and reflects the need for an intersectional lens in yoga research.

Another limitation is that findings from this study may not apply to other regular yoga practitioners, or to other groups of regular AY practitioners, as meaning and knowledge created through qualitative research is situated and contextual. We acknowledge that whilst some yoga practitioners might resonate with our findings, others might not. Additionally, we followed an interpretative approach. While we estimate that our backgrounds and experience with yoga, psychology, and wellbeing science were central in shaping the findings from this study, we also acknowledge that there might be other ways of organizing and interpreting the information gathered in this investigation.

Finally, our review of the yoga traditional and empirical literature, although thorough and rigorous, was not exhaustive by any means. We recognize the complexity of the history and roots of yoga, and have humbly synthesized the most relevant information to provide both breadth and depth to the background, analysis and discussion sections of this article. This study could be followed up by in-depth interviews to further explore this topic.

## Conclusion

Regular yoga practitioners have a complex and nuanced understanding of what yoga means to them, which can open up avenues for discussion, investigation and practice. Yoga was defined as a practice for health and wellbeing, a method for personal inquiry and growth, a way of living, and a spiritual quest. Yoga philosophy definitions intertwined with overall conceptualizations of yoga, and with perceptions of the yoga practice in particular. Yoga philosophy was defined as the collection of ancient teachings to understand human functioning and levels of consciousness, which offered a foundation and support for the yoga practice and living life. Yoga practice was defined as the formal daily AY practice, adaptable, ever-changing, and both permeable to other complementary practices and permeating into daily life. The entanglement of philosophical, spiritual and ethical underpinnings with the practice was noted in three common threads revealing the significance of yoga as an integrated practice, as an embodied path for self-knowledge, and as a path for spiritual development. Thus, yoga appears to be a “*practice of the self”* in which regular practitioners can enhance their understanding of themselves through intra-actions, evolving intrapersonal and inter-relational aspects within themselves, with others, and the world. The prevailing image of yoga as a physical practice for fitness, health and wellbeing seems to be more reflective of societal and scientific trends rather than being representative of regular yoga practitioners. Giving voice to them can contribute in continue shaping the ever changing yoga landscape in our current globalized world.

## Data Availability

Data can be made available by the authors upon request, to be used in closely related projects.
