# Supplementary material for: From practice to lifestyle: conceptualizations of yoga in regular Ashtanga yoga practitioners using reflexive thematic analysis
Source: Front Psychol. 2025 Jun 4;16:1582275. doi: 10.3389/fpsyg.2025.1582275 (PMC12177368; doi:10.3389/fpsyg.2025.1582275)
Supplement: Supplementary file 1 [file Supplementary_file_1.docx]

**Supplement 1.** *Questions Included from the Ashtanga Yoga and Wellbeing Online Survey*

| Topic | Item | Type |
| --- | --- | --- |
| Ashtanga Yoga Practice | | |
| Criterion for selection | Do you consider yourself a regular Ashtanga Yoga practitioner? | Close-ended |
| Years of AY practice | How many years have you been practising Ashtanga Yoga? | Close-ended |
| Years of regular AY practice | How long have you been practising Ashtanga Yoga on a regular basis? | Close-ended |
| AY practice modality | What is your Ashtanga Yoga practice like? Select all that apply | Close-ended |
| Frequency | How many days do you usually practice per week? | Close-ended |
| Duration | How many minutes does your practice usually last, during an average session? | Close-ended |
| Time of the day | At what time do you usually practice? | Close-ended |
| Consistency | In a scale from 0 to 10, with 0 being completely inconsistent and 10 being completely consistent, how consistent is your practice? | Close-ended |
| Asana sequence | What sequence(s) are you currently practising? Select the option that best represents your regular asana practice | Close-ended |
| AY elements | What elements does your Ashtanga Yoga practice include? Select all that apply | Close-ended |
| Ashtanga Yoga Philosophy | | |
| Introduction to questions on eight limbs | Ashtanga Yoga can be translated as the “8 limbs of yoga” and refer not only to the physical practice of yoga, but also to a philosophical framework describing 8 different and interconnected aspects of yoga.  These eight aspects (i.e. yama, niyama, asana, pranayama, pratyahara, dharana, dhyana, samadhi) might be or not a part of your practice, and might vary in terms of their presence.   On a scale of 0 to 10, with 0 being never and 10 being always, please indicate to what extent you consider the presence of each element in your regular practice and/or life: | - |
| Yama | To what extent do you consider and apply moral observances (e.g. non-violence, truthfulness, non-stealing, non-temptation, non-collecting) in your behaviour with others? | Close-ended |
| Niyama | To what extent do you consider and apply moral inner observances (e.g. cleanliness, contentment, self-discipline, self-study, devotion) in your own behaviour? | Close-ended |
| Asana | To what extent do you incorporate physical postures into your own practice? | Close-ended |
| Pranayama | To what extent do you practice yoga breathing techniques? | Close-ended |
| Pratyahara | To what extent do you practice withdrawal from your senses during your practice? | Close-ended |
| Dharana | To what extent do you experience a state of concentration during your practice? | Close-ended |
| Dhyana | To what extent do you experience a state of meditation during your practice? | Close-ended |
| Samadhi | To what extent do you experience a state of bliss, absorption and oneness in your practice? | Close-ended |
| Relevance of philosophy | On a scale from 0 to 10, 0 being completely irrelevant and 10 being completely relevant, how relevant is yoga philosophy to your own practice? | Close-ended |
| Philosophy frequency | How often do you engage with yoga philosophy? | Close-ended |
| Type of yoga philosophy | In which type(s) of yoga philosophy activity do you engage with? Select all that apply | Close-ended |
| Wellbeing Dimensions | | |
| Introduction to wellbeing dimensions | Wellbeing is often defined as comprising of different areas, including but not limited to physical, emotional, psychological, social and spiritual. How would you define each of these dimensions? |  |
| Physical dimension | Physical wellbeing | Open-ended |
| Emotional dimension | Emotional wellbeing | Open-ended |
| Psychological dimension | Psychological wellbeing | Open-ended |
| Social dimension | Social wellbeing | Open-ended |
| Spiritual dimension | Spiritual wellbeing | Open-ended |
| Other dimensions | Any additional area that you consider important and has not been included?  Please comment in the box below which area/s and how you would define it/them: | Open-ended |
| Questions about the relation between AY practice and wellbeing | In a scale from 0 to 10, with 0 being not at all and 10 being completely influenced, to what extent each of the following areas of wellbeing have been positively influenced by your yoga practice? |  |
|  | Physical | Close-ended |
|  | Emotional | Close-ended |
|  | Psychological | Close-ended |
|  | Social | Close-ended |
|  | Spiritual | Close-ended |
| Demographics | | |
| Age | What is your age? | Close-ended |
| Gender | What is your gender? | Close-ended |
| Location | Where do you live? | Close-ended |
| Area | In which are do you live? | Close-ended |
| Citizenship | What is your nationality? | Close-ended |
| Ethnicity | Please specify your ethnicity | Close-ended |
| Education | What is the highest degree or level of education you have completed? | Close-ended |
| Spiritual tradition | With which of the following spiritual or religious traditions do you relate the most? | Close-ended |
|  | Comments | Open-ended |

**Supplement 2.** *Demographic characteristics of the current sample of regular AY practitioners versus the broader set of surveys completed.*

| Characteristic | Category | Regular AY Practitioners  N=200 | | | | Broader Sample  N=352 | | | | |
| --- | --- | --- | --- | --- | --- | --- | --- | --- | --- | --- |
|  |  | N | % | N | | | % | |  |  |
| Gender | Female  Male  Other/Not reported | 159  37  4 | 79.50%  18.50%  2.00% | 253  61  38 | | | 71.88%  17.33%  10.79% | |  |  |
| Age | 18 to 24 years old  25 to 34 years old  35 to 44 years old  45 to 54 years old  55 to 64 years old  65 to 74 years old  75 years old and above  Not reported | 18  62  57  42  19  1  1  0 | 9.00%  31.00%  28.50%  21.00%  9.50%  0.50%  0.50%  0.00% | 25  108  94  69  24  1  1  30 | | | 7.10%  30.68%  26.70%  19.60%  6.82%  0.28%  0.28%  8.52% | |  |  |
| Location | Americas  Europe  Asia  Oceania  Africa  Not reported | 72  52  20  49  2  5 | 32.50%  26.00%  9.50%  24.50%  1.00%  2.50% | 120  88  36  69  2  37 | | | 30.11%  23.58%  6.25%  19.60%  0.57%  10.51% | |  |  |
| Area | Urban  Sub-urban  Rural  Other/Not reported | 132  47  17  4 | 66.00%  23.50%  8.50%  2.00% | 204  79  32  37 | | | 57.95%  22.44%  9.09%  10.51% | |  |  |
| Citizenship | Same as location  Different from location  Not reported | 148  46  6 | 74.00%  23.00%  3.00% | 250  70  32 | | | 71.02%  19.89%  9.09% | |  |  |
| Education | High school education incomplete  High school education complete  Trade vocational  Bachelor  Master  Doctorate  Not reported | 2  11  16  77  70  24  0 | 1.00%  5.50%  8.00%  38.50%  35.00%  12.00%  0.00% | 6  16  24  131  110  33  32 | | | 1.70%  4.55%  6.82%  37.22%  31.25%  9.38%  9.09% | |  |  |
| Survey Language | English  Spanish | 173  27 | 86.50%  13.50% | 302  50 | | | 85.80%  14.20% | |  |  |
| Ethnicity | Arabic  African  Asian  Caucasian  Hispanic or Latino  Native American  Multiethnic  Other/Not reported | 1  1  17  115  30  2  22  12 | 0.50%  0.50%  8.50%  57.50%  15.00%  1.00%  11.00%  6.00% | 1  2  33  182  48  8  29  49 | | | 0.28%  0.57%  9.38%  51.70%  13.64%  2.27%  8.24%  13.92% | |  |  |
| Spiritual Tradition | Buddhism  Christianity  Hinduism  Islam  Judaism  Atheism  Agnosticism  Aboriginal Spirituality  None of the above  Other/Not reported | 38  32  14  2  3  28  18  1  54  10 | 19.00%  16.00%  7.00%  1.00%  1.50%  14.00%  9.00%  0.50%  27.00%  5.00% | | 60  63  20  2  3  44  29  2  73  56 | | | 17.05%  17.90%  5.68%  0.57%  0.85%  12.50%  8.24%  0.57%  20.74%  15.91% | |  |

**Supplement 3.** *Characteristics of regular Ashtanga Yoga practitioners’ (RAYP) yoga practice versus the broader sample of participants*

| Aspect of AY practice | Category | RAYP  N=200 | | Broader Sample  N=352 | |
| --- | --- | --- | --- | --- | --- |
|  |  | N | % | N | % |
| Years of AY practice | Less than 3 months  3 to 6 months  6 months to 12 months  1 to 2 years  3 to 5 years  6 to 10 years  11 to 15 years  16 to 20 years  More than 20 years  Not reported | 2  4  13  34  61  38  23  19  6  0 | 1.00%  2.00%  6.50%  17.00%  30.50%  19.00%  11.50%  9.50%  3.00%  0.00% | 14  9  21  53  84  52  27  27  11  54 | 3.98%  2.56%  5.97%  15.06%  23.86%  14.77%  7.67%  7.67%  3.13%  15.34% |
| Years of regular AY practice | Less than 3 months  3 to 6 months  6 months to 12 months  1 to 2 years  3 to 5 years  6 to 10 years  11 to 15 years  16 to 20 years  More than 20 years  Not reported | 4  9  16  40  57  39  15  14  4  2 | 2.00%  4.50%  8.00%  20.00%  28.50%  19.50%  7.50%  7.00%  2.00%  1.00% | 4  12  21  47  66  44  16  19  4  119 | 1.14%  3.41%  5.97%  13.35%  18.75%  12.50%  4.55%  5.40%  1.14%  33.81% |
| AY practice modality | Mysore style  Traditional Sanskrit-led class  Led-class (primary or intermediate)  Guided AY based class (different each class)  Self-practice  Mysore and traditional Sanskrit-led class  Mysore and led class  Mysore and guided AY based class  Mysore, Sanskrit-led and led-class  Mysore, Sanskrit-led and guided AY based  Mysore, Sanskrit-led, led-class and guided  Mysore, led-class and guided AY based  Mysore and Self-practice  Sanskrit-led and led class  Sanskrit-led and guided AY based class  Led-class and guided AY based class  Sanskrit-led, led-class and guided AY based  Other  Not reported | 89  4  2  5  3  49  12  1  22  1  5  4  1  0  0  0  1  1  0 | 44.50%  2.00%  1.00%  2.50%  1.50%  24.50%  6.00%  0.50%  11.00%  0.50%  2.50%  2.00%  0.50%  0.00%  0.00%  0.00%  0.50%  0.50%  0.00% | 122  7  8  13  3  69  18  2  27  3  7  8  1  2  1  1  2  3  55 | 34.66%  1.99%  2.27%  3.69%  0.85%  19.60%  5.11%  0.57%  7.67%  0.85%  1.99%  2.27%  0.28%  0.57%  0.28%  0.28%  0.57%  0.85%  15.63% |
| Frequency | Less than once a week  1 to 2 days per week  3 to 4 days per week  5 to 6 days per week  Everyday  It depends  Not reported | 0  13  49  108  7  21  2 | 0.00%  6.50%  24.50%  54.00%  3.50%  10.50%  1.00% | 5  38  78  135  13  27  56 | 1.42%  10.80%  22.16%  38.35%  3.69%  7.67%  15.91% |
| Duration | Less than 20 minutes  20 to 40 minutes  41 to 60 minutes  61 to 80 minutes  81 to 100 minutes  101 to 120 minutes  More than 120 minutes  It depends  Not reported | 0  3  20  62  56  43  14  2  0 | 0.00%  1.50%  10.00%  31.00%  28.00%  21.50%  7.00%  1.00%  0.00% | 4  8  39  90  83  52  17  5  54 | 1.14%  2.27%  11.08%  25.57%  23.58%  14.77%  4.83%  1.42%  15.34% |
| Time of the day | 6am to 10am  11am to 1pm  2pm to 5pm  6pm to 9pm  It depends  Not reported | 127  13  7  22  31  0 | 63.50%  6.50%  3.50%  11.00%  15.50%  0.00% | 178  20  12  44  44  54 | 50.57%  5.68%  3.41%  12.50%  12.50%  15.34% |
| Consistency | 1  2  3  4  5  6  7  8  9  10  Not reported | 0  1  0  3  2  20  52  59  37  24  2 | 0.00%  0.50%  0.00%  1.50%  0.50%  10.00%  26.00%  29.50%  18.50%  12.00%  1.00% | 3  6  9  11  15  32  64  74  43  35  60 | 0.85%  1.70%  2.56%  3.13%  4.26%  9.09%  18.18%  21.02%  12.22%  9.94%  17.05% |
| Asana sequence | Surya Namaskar A and B  Surya Namaskar and standing  Primary series up to Janu C  Primary series up to Navasana  Second half of Primary series  Full Primary series  Primary up to 1^st^ half of Intermediate  Primary up to 2^nd^ half of Intermediate  Full Primary and Intermediate  Primary, Int. up to 1^st^ half of Adv. A  Primary, Int. up to 2^nd^ half of Adv. A  Full Primary, Int. and Adv. A  Full Primary, Int. and Adv. A and B  Other  Not reported | 0  1  3  28  6  53  63  8  14  6  1  2  2  13  0 | 0.00%  0.50%  1.50%  14.00%  3.00%  26.50%  31.50%  4.00%  7.00%  3.00%  0.50%  1.00%  1.00%  6.50%  0.00% | 6  4  6  54  9  81  81  10  15  8  1  3  2  16  56 | 1.70%  1.14%  1.70%  15.34%  2.56%  23.01%  23.01%  2.84%  4.26%  2.27%  0.28%  0.85%  0.57%  4.55%  15.91% |
| AY elements | Free breathing with sound  Use of bandhas  Use of drishti  Chanting opening and closing mantras  Meditation before or after practice  Pranayama before or after practice  Resting on moon days  Vinyasa throughout practice  Other  Not reported | 182  181  187  173  94  109  135  192  13  0 | 91.00%  90.50%  93.50%  86.50%  47.00%  54.50%  67.50%  96.00%  6.50%  0.00% | 253  251  266  244  139  124  190  276  18  54 | 71.88%  71.31%  75.57%  69.32%  39.49%  35.23%  53.98%  78.41%  5.11%  15.34% |

**Supplement 4.** *Regular* *AYPs’ engagement in Ashtanga Yoga Philosophy*

| Aspect of Yoga Philosophy | Category | RAYP  N=200 | | | Broader Sample  N=352 | |
| --- | --- | --- | --- | --- | --- | --- |
|  |  | N | % | N | | % |
| Yama | 0  1  2  3  4  5  6  7  8  9  10  Not reported | 1  1  0  2  2  14  10  52  61  32  21  4 | 0.50%  0.50%  0.00%  1.00%  1.00%  7.00%  5.00%  26.00%  30.50%  16.00%  10.50%  2.00% | 1  2  2  3  2  22  17  63  79  46  31  84 | | 0.28%  0.57%  0.57%  0.85%  0.57%  6.25%  4.83%  17.90%  22.44%  13.07%  8.81%  23.86% |
| Niyama | 0  1  2  3  4  5  6  7  8  9  10  Not reported | 1  0  1  2  8  12  24  51  62  22  12  5 | 0.50%  0.00%  0.50%  1.00%  4.00%  6.00%  12.00%  25.50%  31.00%  11.00%  6.00%  2.50% | 1  0  2  4  12  19  34  67  77  31  18  87 | | 0.28%  0.00%  0.57%  1.14%  3.41%  5.40%  9.66%  19.03%  21.88%  8.81%  5.11%  24.72% |
| Asana | 0  1  2  3  4  5  6  7  8  9  10  Not reported | 1  0  1  2  8  12  24  51  62  22  12  5 | 0.50%  0.00%  0.50%  1.00%  4.00%  6.00%  12.00%  25.50%  31.00%  11.00%  6.00%  2.50% | 0  1  0  1  3  12  6  21  46  56  120  86 | | 0.00%  0.28%  0.00%  0.28%  0.85%  3.41%  1.70%  5.97%  13.07%  15.91%  34.09%  24.43% |
| Pranayama | 0  1  2  3  4  5  6  7  8  9  10  Not reported | 0  1  1  3  4  9  15  22  48  34  59  4 | 0.00%  0.50%  0.50%  1.50%  2.00%  4.50%  7.50%  11.00%  24.00%  17.00%  29.50%  2.00% | 1  2  3  6  6  19  18  25  64  47  77  84 | | 0.28%  0.57%  0.85%  1.70%  1.70%  5.40%  5.11%  7.10%  18.18%  13.35%  21.88%  23.86% |
| Pratyahara | 0  1  2  3  4  5  6  7  8  9  10  Not reported | 3  3  10  12  16  27  25  40  32  15  8  9 | 1.50%  1.50%  5.00%  6.00%  8.00%  13.50%  12.50%  20.00%  16.00%  7.50%  4.00%  4.50% | 6  7  17  13  20  37  35  50  41  21  12  93 | | 1.70%  1.99%  4.83%  3.69%  5.68%  10.51%  9.94%  14.20%  11.65%  5.97%  3.41%  26.42% |
| Dharana | 0  1  2  3  4  5  6  7  8  9  10  Not reported | 0  0  0  1  2  19  27  47  62  26  10  6 | 0.00%  0.00%  0.00%  0.50%  1.00%  9.50%  13.50%  23.50%  31.00%  13.00%  5.00%  3.00% | 0  2  1  3  7  24  33  58  83  36  18  87 | | 0.00%  0.57%  0.28%  0.85%  1.99%  6.82%  9.38%  16.48%  23.58%  10.23%  5.11%  24.72% |
| Dhyana | 0  1  2  3  4  5  6  7  8  9  10  Not reported | 1  3  5  10  6  24  33  49  37  19  7  6 | 0.50%  1.50%  2.50%  5.00%  3.00%  12.00%  16.50%  24.50%  18.50%  9.50%  3.50%  3.00% | 5  6  7  14  9  33  45  59  49  25  12  88 | | 1.42%  1.70%  1.99%  3.98%  2.57%  9.38%  12.78%  16.76%  13.92%  7.10%  3.41%  25.00% |
| Samadhi | 0  1  2  3  4  5  6  7  8  9  10  Not reported | 3  6  10  19  17  27  21  30  39  15  8  5 | 1.50%  3.00%  5.00%  9.50%  8.50%  13.50%  10.50%  15.00%  19.50%  7.50%  4.00%  2.50% | 5  10  12  26  24  33  31  38  51  20  13  89 | | 1.42%  1.70%  3.41%  7.39%  6.82%  9.38%  8.81%  10.80%  14.49%  5.68%  3.69%  25.28% |
| Relevance of philosophy | 0  1  2  3  4  5  6  7  8  9  10  Not reported | 1  3  3  4  6  13  16  25  37  21  66  5 | 0.50%  1.50%  1.50%  2.00%  3.00%  6.50%  8.00%  12.50%  18.50%  10.50%  33.00%  2.50% | 2  3  6  7  11  16  23  35  54  29  78  88 | | 0.57%  0.85%  1.70%  1.99%  3.13%  4.55%  6.53%  9.94%  15.34%  8.24%  22.16%  25.00% |
| Philosophy frequency | Never  Less than once per month  Once or twice per month  Once or twice per week  Almost daily  Not reported | 5  32  63  50  46  4 | 2.50%  16.00%  31.50%  25.00%  23.00%  2.00% | 12  44  58  70  83  85 | | 3.41%  12.50%  16.48%  19.89%  23.58%  24.15% |
| Type of yoga philosophy | After class conference by teacher  Study and chanting of mantras  Yoga teacher training programs  Yoga retreats  Public events  Face to face courses  Online courses  Reading books and texts  Online videos  Reading blogs  Reading social media posts  Other  Not reported | 82  89  67  57  32  51  109  162  98  100  102  14  9 | 41.00%  44.50%  33.50%  28.50%  16.00%  25.50%  54.50%  81.00%  49.00%  50.00%  51.00%  7.00%  4.50% | 97  119  80  73  43  61  63  208  124  125  126  18  100 | | 27.57%  33.81%  22.73%  20.74%  12.22%  17.33%  17.90%  59.09%  35.23%  35.51%  35.80%  5.11%  28.41% |

**Supplement 5.** *Perceived Influence of Yoga Practice on Different Dimensions of Regular AYPs’ Wellbeing*

| Wellbeing Dimension | Category | RAYP  N=200 | | | Broader Sample  N=352 | |
| --- | --- | --- | --- | --- | --- | --- |
|  |  | N | % | N | | % |
| Physical | 0  1  2  3  4  5  6  7  8  9  10  Not reported | 1  0  0  0  1  1  2  4  16  25  94  56 | 0.50%  0.00%  0.00%  0.00%  0.50%  0.50%  1.00%  2.00%  8.00%  12.50%  47.00%  28.00% | 1  1  1  0  1  3  4  10  23  36  109  163 | | 0.28%  0.28%  0.28%  0.00%  0.28%  0.85%  1.14%  2.84%  6.53%  10.23%  30.97%  46.31% |
| Emotional | 0  1  2  3  4  5  6  7  8  9  10  Not reported | 1  0  0  0  1  0  4  21  23  24  69  57 | 0.50%  0.00%  0.00%  0.00%  0.50%  0.00%  2.00%  10.50%  11.50%  12.00%  34.50%  28.50% | 1  1  0  0  1  1  6  23  35  31  87  166 | | 0.28%  0.28%  0.00%  0.00%  0.28%  0.28%  1.70%  6.53%  9.94%  8.81%  24.71%  47.16% |
| Psychological | 0  1  2  3  4  5  6  7  8  9  10  Not reported | 1  0  1  1  0  1  6  9  29  22  71  59 | 0.50%  0.00%  0.50%  0.50%  0.00%  0.50%  3.00%  4.50%  14.50%  11.00%  35.50%  29.50% | 1  2  1  0  1  2  8  11  39  31  87  169 | | 0.28%  0.57%  0.28%  0.00%  0.28%  0.57%  2.27%  3.13%  11.08%  8.81%  24.72%  48.01% |
| Social | 0  1  2  3  4  5  6  7  8  9  10  Not reported | 1  1  1  5  3  10  21  22  23  17  35  61 | 0.50%  0.50%  0.50%  2.50%  1.50%  5.00%  10.50%  11.00%  11.50%  8.50%  17.50%  30.50% | 1  2  5  6  6  14  26  28  34  17  43  170 | | 0.28%  0.57%  1.42%  1.70%  1.70%  3.98%  7.39%  7.95%  9.66%  4.83%  12.22%  48.30% |
| Spiritual | 0  1  2  3  4  5  6  7  8  9  10  Not reported | 1  1  0  3  1  3  5  11  23  19  70  63 | 0.50%  0.50%  0.00%  1.50%  0.50%  1.50%  2.50%  10.50%  12.50%  9.50%  35.00%  31.50% | 1  2  0  3  4  7  6  13  30  28  83  175 | | 0.28%  0.57%  0.00%  0.85%  1.14%  1.99%  1.70%  3.69%  8.52%  7.95%  23.58%  49.72% |

**Supplement 6.** *Perceived Levels of Wellbeing Dimensions in Regular AYPs*

| Wellbeing Dimension | Category | RAYP  N=137* | | | Broader Sample  N=352 | |
| --- | --- | --- | --- | --- | --- | --- |
|  |  | N | % | N | | % |
| Physical | 0  1  2  3  4  5  6  7  8  9  10  Not reported | 0  0  0  0  1  2  6  28  40  39  18  3 | 0.00%  0.00%  0.00%  0.00%  0.73%  1.46%  4.38%  20.44%  29.20%  28.47%  13.14%  2.19% | 1  0  0  2  2  6  12  36  56  47  27  163 | | 0.28%  0.00%  0.00%  0.57%  0.57%  1.70%  3.41%  10.23%  15.91%  13.35%  7.67%  46.31% |
| Emotional | 0  1  2  3  4  5  6  7  8  9  10  Not reported | 0  0  1  2  1  10  9  37  43  22  10  2 | 0.00%  0.00%  0.73%  1.46%  0.73%  7.30%  6.57%  27.01%  31.39%  16.06%  7.30%  1.46% | 0  0  1  2  2  15  16  56  54  25  18  163 | | 0.00%  0.00%  0.28%  0.57%  0.57%  4.26%  4.55%  15.91%  15.34%  7.10%  5.11%  46.31% |
| Psychological | 0  1  2  3  4  5  6  7  8  9  10  Not reported | 0  0  1  2  3  10  12  36  37  19  12  5 | 0.00%  0.00%  0.73%  1.46%  2.19%  7.30%  8.76%  26.28%  27.01%  13.87%  8.76%  3.65% | 0  0  1  2  5  14  19  48  50  25  22  166 | | 0.00%  0.00%  0.28%  0.57%  1.42%  3.98%  5.40%  13.64%  14.20%  7.10%  6.25%  47.16% |
| Social | 0  1  2  3  4  5  6  7  8  9  10  Not reported | 1  1  2  1  5  17  16  26  30  22  11  5 | 0.73%  0.73%  1.46%  0.73%  3.65%  12.41%  11.68%  18.98%  21.90%  16.06%  8.03%  3.65% | 2  1  2  5  6  24  23  34  45  23  19  168 | | 0.57%  0.28%  0.57%  1.42%  1.70%  6.82%  6.53%  9.66%  12.78%  6.53%  5.40%  47.73% |
| Spiritual | 0  1  2  3  4  5  6  7  8  9  10  Not reported | 0  0  0  2  3  10  19  22  29  22  21  9 | 0.00%  0.00%  0.00%  1.46%  2.19%  7.30%  13.87%  16.06%  21.17%  16.06%  15.33%  6.57% | 1  0  0  2  5  17  26  36  37  25  31  172 | | 0.28%  0.00%  0.00%  0.57%  1.42%  4.83%  7.39%  10.23%  10.51%  7.10%  8.81%  48.86% |

*only 137 of 200 participants completed these questions

**Supplement 7.** *Word Frequency of Words Included in Yoga Word Cloud*

| Word | Length | Count | Weighted Percentage |
| --- | --- | --- | --- |
| yoga | 4 | 125 | 4.58% |
| life | 4 | 73 | 2.67% |
| mind | 4 | 53 | 1.94% |
| way | 3 | 47 | 1.72% |
| body | 4 | 38 | 1.39% |
| practice | 8 | 35 | 1.28% |
| means | 5 | 32 | 1.17% |
| physical | 8 | 30 | 1.10% |
| self | 4 | 25 | 0.92% |
| time | 4 | 18 | 0.66% |
| better | 6 | 17 | 0.62% |
| world | 5 | 16 | 0.59% |
| also | 4 | 15 | 0.55% |
| feel | 4 | 15 | 0.55% |
| helps | 5 | 15 | 0.55% |
| living | 6 | 14 | 0.51% |
| union | 5 | 14 | 0.51% |
| back | 4 | 13 | 0.48% |
| different | 9 | 13 | 0.48% |
| peace | 5 | 13 | 0.48% |
| everything | 10 | 12 | 0.44% |
| moment | 6 | 12 | 0.44% |
| one | 3 | 12 | 0.44% |
| breath | 6 | 11 | 0.40% |
| get | 3 | 11 | 0.40% |
| health | 6 | 11 | 0.40% |
| like | 4 | 11 | 0.40% |
| lot | 3 | 11 | 0.40% |
| mental | 6 | 11 | 0.40% |
| part | 4 | 11 | 0.40% |
| asana | 5 | 10 | 0.37% |
| connection | 10 | 10 | 0.37% |
| daily | 5 | 10 | 0.37% |
| see | 3 | 10 | 0.37% |
| something | 9 | 10 | 0.37% |
| spiritual | 9 | 10 | 0.37% |
| state | 5 | 10 | 0.37% |
| allows | 6 | 9 | 0.33% |
| form | 4 | 9 | 0.33% |
| helped | 6 | 9 | 0.33% |
| inner | 5 | 9 | 0.33% |
| makes | 5 | 9 | 0.33% |
| people | 6 | 9 | 0.33% |
| really | 6 | 9 | 0.33% |
| things | 6 | 9 | 0.33% |
| wellbeing | 9 | 9 | 0.33% |
| without | 7 | 9 | 0.33% |
| discipline | 10 | 8 | 0.29% |
| every | 5 | 8 | 0.29% |
| focus | 5 | 8 | 0.29% |
| lifestyle | 9 | 8 | 0.29% |
| meditation | 10 | 8 | 0.29% |
| others | 6 | 8 | 0.29% |
| think | 5 | 8 | 0.29% |
| want | 4 | 8 | 0.29% |
| around | 6 | 7 | 0.26% |
| calm | 4 | 7 | 0.26% |
| changed | 7 | 7 | 0.26% |
| find | 4 | 7 | 0.26% |
| human | 5 | 7 | 0.26% |
| place | 5 | 7 | 0.26% |
| present | 7 | 7 | 0.26% |
| question | 8 | 7 | 0.26% |
| soul | 4 | 7 | 0.26% |
| tool | 4 | 7 | 0.26% |
| true | 4 | 7 | 0.26% |
| well | 4 | 7 | 0.26% |
| answer | 6 | 6 | 0.22% |
| ashtanga | 8 | 6 | 0.22% |
| awareness | 9 | 6 | 0.22% |
| day | 3 | 6 | 0.22% |
| experience | 10 | 6 | 0.22% |
| gives | 5 | 6 | 0.22% |
| good | 4 | 6 | 0.22% |
| just | 4 | 6 | 0.22% |
| knowledge | 9 | 6 | 0.22% |
| limbs | 5 | 6 | 0.22% |
| mat | 3 | 6 | 0.22% |
| much | 4 | 6 | 0.22% |
| now | 3 | 6 | 0.22% |
| path | 4 | 6 | 0.22% |
| take | 4 | 6 | 0.22% |
| teacher | 7 | 6 | 0.22% |
| thoughts | 8 | 6 | 0.22% |
| training | 8 | 6 | 0.22% |
| understand | 10 | 6 | 0.22% |
| able | 4 | 5 | 0.18% |
| always | 6 | 5 | 0.18% |
| connect | 7 | 5 | 0.18% |
| conscious | 9 | 5 | 0.18% |
| course | 6 | 5 | 0.18% |
| deal | 4 | 5 | 0.18% |
| emotional | 9 | 5 | 0.18% |
| everyday | 8 | 5 | 0.18% |
| first | 5 | 5 | 0.18% |
| improve | 7 | 5 | 0.18% |
| keep | 4 | 5 | 0.18% |
| know | 4 | 5 | 0.18% |
| let | 3 | 5 | 0.18% |
| level | 5 | 5 | 0.18% |

**Supplement 8.** *Word Frequency of Words Included in Yoga Philosophy Word Cloud*

| **Word** | **Length** | **Count** | **Weighted Percentage** |
| --- | --- | --- | --- |
| yoga | 4 | 116 | 4.86% |
| practice | 8 | 69 | 2.89% |
| life | 4 | 65 | 2.72% |
| philosophy | 10 | 58 | 2.43% |
| way | 3 | 42 | 1.76% |
| mind | 4 | 33 | 1.38% |
| body | 4 | 32 | 1.34% |
| live | 4 | 20 | 0.84% |
| living | 6 | 19 | 0.80% |
| means | 5 | 19 | 0.80% |
| self | 4 | 18 | 0.75% |
| find | 4 | 14 | 0.59% |
| spiritual | 9 | 14 | 0.59% |
| understanding | 13 | 14 | 0.59% |
| also | 4 | 13 | 0.54% |
| limbs | 5 | 13 | 0.54% |
| important | 9 | 12 | 0.50% |
| know | 4 | 12 | 0.50% |
| one | 3 | 12 | 0.50% |
| others | 6 | 12 | 0.50% |
| think | 5 | 12 | 0.50% |
| asana | 5 | 11 | 0.46% |
| ashtanga | 8 | 11 | 0.46% |
| feel | 4 | 11 | 0.46% |
| just | 4 | 11 | 0.46% |
| physical | 8 | 11 | 0.46% |
| without | 7 | 11 | 0.46% |
| world | 5 | 11 | 0.46% |
| love | 4 | 10 | 0.42% |
| path | 4 | 10 | 0.42% |
| peace | 5 | 10 | 0.42% |
| much | 4 | 9 | 0.38% |
| part | 4 | 9 | 0.38% |
| daily | 5 | 8 | 0.33% |
| everything | 10 | 8 | 0.33% |
| guide | 5 | 8 | 0.33% |
| looking | 7 | 8 | 0.33% |
| mat | 3 | 8 | 0.33% |
| niyamas | 7 | 8 | 0.33% |
| sutras | 6 | 8 | 0.33% |
| things | 6 | 8 | 0.33% |
| understand | 10 | 8 | 0.33% |
| yamas | 5 | 8 | 0.33% |
| aspects | 7 | 7 | 0.29% |
| connected | 9 | 7 | 0.29% |
| experience | 10 | 7 | 0.29% |
| helps | 5 | 7 | 0.29% |
| journey | 7 | 7 | 0.29% |
| knowledge | 9 | 7 | 0.29% |
| lifestyle | 9 | 7 | 0.29% |
| like | 4 | 7 | 0.29% |
| people | 6 | 7 | 0.29% |
| time | 4 | 7 | 0.29% |
| towards | 7 | 7 | 0.29% |
| asanas | 6 | 6 | 0.25% |
| aware | 5 | 6 | 0.25% |
| behind | 6 | 6 | 0.25% |
| compassion | 10 | 6 | 0.25% |
| guidance | 8 | 6 | 0.25% |
| learning | 8 | 6 | 0.25% |
| little | 6 | 6 | 0.25% |
| mental | 6 | 6 | 0.25% |
| nature | 6 | 6 | 0.25% |
| practise | 8 | 6 | 0.25% |
| see | 3 | 6 | 0.25% |
| study | 5 | 6 | 0.25% |
| studying | 8 | 6 | 0.25% |
| try | 3 | 6 | 0.25% |
| trying | 6 | 6 | 0.25% |
| wisdom | 6 | 6 | 0.25% |
| work | 4 | 6 | 0.25% |
| acceptance | 10 | 5 | 0.21% |
| around | 6 | 5 | 0.21% |
| change | 6 | 5 | 0.21% |
| eight | 5 | 5 | 0.21% |
| general | 7 | 5 | 0.21% |
| gives | 5 | 5 | 0.21% |
| health | 6 | 5 | 0.21% |
| human | 5 | 5 | 0.21% |
| learn | 5 | 5 | 0.21% |
| meaning | 7 | 5 | 0.21% |
| meditation | 10 | 5 | 0.21% |
| non | 3 | 5 | 0.21% |
| patanjali | 9 | 5 | 0.21% |
| practices | 9 | 5 | 0.21% |
| present | 7 | 5 | 0.21% |
| provides | 8 | 5 | 0.21% |
| question | 8 | 5 | 0.21% |
| really | 6 | 5 | 0.21% |
| seek | 4 | 5 | 0.21% |
| spirit | 6 | 5 | 0.21% |
| traditions | 10 | 5 | 0.21% |
| truth | 5 | 5 | 0.21% |
| able | 4 | 4 | 0.17% |
| answer | 6 | 4 | 0.17% |
| associated | 10 | 4 | 0.17% |
| awareness | 9 | 4 | 0.17% |
| back | 4 | 4 | 0.17% |
| beings | 6 | 4 | 0.17% |
| best | 4 | 4 | 0.17% |

**Supplement 9.** *Word Frequency of Words Included in Yoga Practice Word Cloud*

| **Word** | **Length** | **Count** | **Weighted Percentage** |
| --- | --- | --- | --- |
| practice | 8 | 225 | 5.44% |
| week | 4 | 68 | 1.64% |
| primary | 7 | 63 | 1.52% |
| yoga | 4 | 60 | 1.45% |
| ashtanga | 8 | 54 | 1.31% |
| series | 6 | 54 | 1.31% |
| day | 3 | 44 | 1.06% |
| days | 4 | 44 | 1.06% |
| body | 4 | 37 | 0.90% |
| asana | 5 | 36 | 0.87% |
| pranayama | 9 | 35 | 0.85% |
| meditation | 10 | 34 | 0.82% |
| mysore | 6 | 30 | 0.73% |
| time | 4 | 30 | 0.73% |
| sometimes | 9 | 25 | 0.60% |
| full | 4 | 24 | 0.58% |
| teacher | 7 | 24 | 0.58% |
| times | 5 | 24 | 0.58% |
| intermediate | 12 | 23 | 0.56% |
| morning | 7 | 23 | 0.56% |
| feel | 4 | 22 | 0.53% |
| home | 4 | 21 | 0.51% |
| breathing | 9 | 20 | 0.48% |
| life | 4 | 20 | 0.48% |
| start | 5 | 20 | 0.48% |
| work | 4 | 20 | 0.48% |
| years | 5 | 20 | 0.48% |
| daily | 5 | 18 | 0.44% |
| mantra | 6 | 18 | 0.44% |
| one | 3 | 18 | 0.44% |
| shala | 5 | 18 | 0.44% |
| try | 3 | 18 | 0.44% |
| practicing | 10 | 17 | 0.41% |
| usually | 7 | 17 | 0.41% |
| minutes | 7 | 16 | 0.39% |
| now | 3 | 16 | 0.39% |
| also | 4 | 15 | 0.36% |
| asanas | 6 | 15 | 0.36% |
| classes | 7 | 15 | 0.36% |
| rest | 4 | 15 | 0.36% |
| sequence | 8 | 15 | 0.36% |
| always | 6 | 14 | 0.34% |
| led | 3 | 14 | 0.34% |
| poses | 5 | 14 | 0.34% |
| just | 4 | 13 | 0.31% |
| style | 5 | 13 | 0.31% |
| yin | 3 | 13 | 0.31% |
| energy | 6 | 12 | 0.29% |
| every | 5 | 12 | 0.29% |
| followed | 8 | 12 | 0.29% |
| get | 3 | 12 | 0.29% |
| half | 4 | 12 | 0.29% |
| like | 4 | 12 | 0.29% |
| mind | 4 | 12 | 0.29% |
| postures | 8 | 12 | 0.29% |
| since | 5 | 12 | 0.29% |
| sun | 3 | 12 | 0.29% |
| back | 4 | 11 | 0.27% |
| chant | 5 | 11 | 0.27% |
| first | 5 | 11 | 0.27% |
| moment | 6 | 11 | 0.27% |
| standing | 8 | 11 | 0.27% |
| study | 5 | 11 | 0.27% |
| two | 3 | 11 | 0.27% |
| vinyasa | 7 | 11 | 0.27% |
| well | 4 | 11 | 0.27% |
| end | 3 | 10 | 0.24% |
| much | 4 | 10 | 0.24% |
| opening | 7 | 10 | 0.24% |
| second | 6 | 10 | 0.24% |
| chanting | 8 | 9 | 0.22% |
| closing | 7 | 9 | 0.22% |
| currently | 9 | 9 | 0.22% |
| good | 4 | 9 | 0.22% |
| keep | 4 | 9 | 0.22% |
| mat | 3 | 9 | 0.22% |
| mostly | 6 | 9 | 0.22% |
| physical | 8 | 9 | 0.22% |
| self | 4 | 9 | 0.22% |
| started | 7 | 9 | 0.22% |
| surya | 5 | 9 | 0.22% |
| breath | 6 | 8 | 0.19% |
| challenging | 11 | 8 | 0.19% |
| covid | 5 | 8 | 0.19% |
| everyday | 8 | 8 | 0.19% |
| feeling | 7 | 8 | 0.19% |
| need | 4 | 8 | 0.19% |
| others | 6 | 8 | 0.19% |
| per | 3 | 8 | 0.19% |
| practise | 8 | 8 | 0.19% |
| seated | 6 | 8 | 0.19% |
| working | 7 | 8 | 0.19% |
| almost | 6 | 7 | 0.17% |
| around | 6 | 7 | 0.17% |
| finish | 6 | 7 | 0.17% |
| meditate | 8 | 7 | 0.17% |
| moon | 4 | 7 | 0.17% |
| namaskar | 8 | 7 | 0.17% |
| part | 4 | 7 | 0.17% |
| philosophy | 10 | 7 | 0.17% |

**Supplement 10.** *Word tree of the word feel for Yoga*


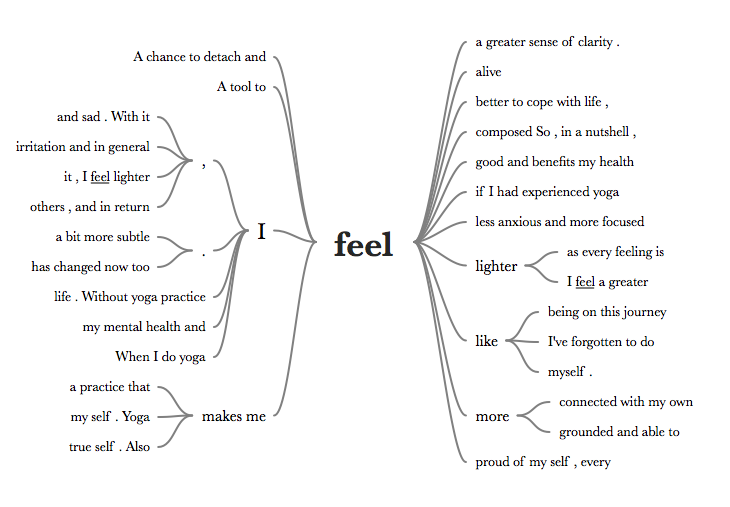


**Supplement 11.** *Word tree of the word feel for Yoga Philosophy*


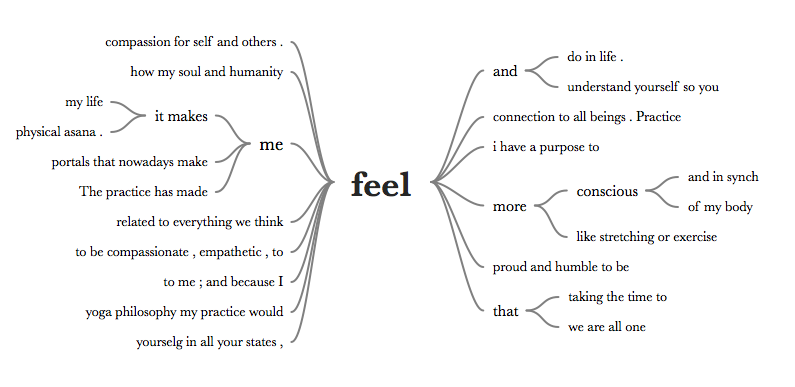


**Supplement 12.** *Word tree of the word feel for Yoga*


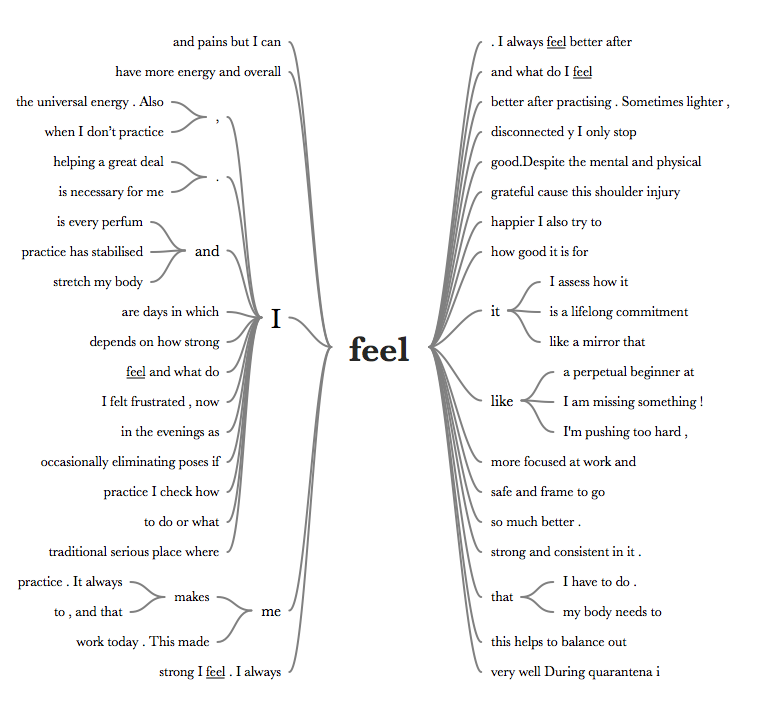


**Supplement 13.** *Summary of Themes and Sub-themes for Each Aspect of Yoga*

| Yoga Aspect | Themes | Sub-themes | N | | |
| --- | --- | --- | --- | --- | --- |
| Yoga | *Yoga is a holistic and multidimensional practice* | A physical practice | 20 | | |
|  |  | A mind-body practice | 37 | | |
|  |  | A contemplative practice | 26 | | |
|  |  | A spiritual practice | 33 | | |
|  | *Yoga is a tool for healing, coping and cultivating health and wellbeing* | A healing tool | 15 | | |
|  |  | A coping tool for life challenges | 11 | | |
|  |  | A tool for balancing and improving health and wellbeing | 28 | | |
|  | *Yoga is a method for knowing, accepting and developing the self* | A method for self-development and self-transformation | 24 | | |
|  |  | A method for self-inquiry and self-discovery | 37 | | |
|  |  | A system of self-care and self-discipline | 13 | | |
|  | *Yoga is a way of being, living and seeing the world* | A way of connecting with others and having a sense of community | 15 | | |
|  |  | A way of grounding, centring and calming | 35 | | |
|  |  | A way of living and perceiving the world | 58 | | |
|  | *Yoga is the journey towards the realisation of the Self* | A way to steady the mind | 25 | | |
|  |  | Experiencing or practising a state of union or oneness | 33 | | |
|  |  | The realisation of the true self | 14 | | |
|  |  | The state and practice of clarity | 4 | | |
| Yoga Philosophy | *A collection of teachings for the knowledge and evolution of the self* | A path for the evolution of the self | 15 | | |
|  |  | Cultivation of awareness and observance | 9 | | |
|  |  | Learning to connect, accept and understand oneself | 19 | | |
|  | *An applied framework for the yoga asana practice and daily life* | A framework to understand and support the practice | 23 | | |
|  |  | A way of being, thinking and interacting in the world | 41 | | |
|  |  | A way of living | 27 | | |
|  |  | Embodying the Yoga Sutras in yoga practice and life | 21 | | |
|  | *A spiritual path towards the understanding of reality, human nature and beyond* | Embracing impermanence | 5 | | |
|  |  | Steadiness of the mind | 6 | | |
|  |  | The interconnection of body, mind and spirit | 14 | | |
|  |  | Understanding the nature of the self and the spiritual journey to the Self | 14 | | |
|  | *A spiritual path towards the understanding of reality, human nature and beyond* | Drawing connections to other philosophies or spiritual traditions | 8 | | |
|  |  | Understanding the foundations, teachings and background of the practice | 24 | | |
|  |  | Worldviews intertwined with practices | 14 | | |
|  | *Attitudes and engagement towards Yoga Philosophy* | A minimal engagement with the philosophy | 14 | | |
|  |  | Humbled by yoga philosophy | 7 | | |
|  |  | Self-study | 10 | | |
| Yoga Practice | *Description of the elements included in the yoga practice* | AY asana sequence | 100 |  |  |
|  |  | AY mantras | 14 |  |  |
|  |  | Informal practice | 21 |  |  |
|  |  | Meditation | 38 |  |  |
|  |  | Mysore style | 18 |  |  |
|  |  | Pranayama | 41 |  |  |
|  |  | Resting days | 7 |  |  |
|  |  | Self-study | 13 |  |  |
|  |  | Vedic chanting | 8 | |  |
|  | *Outcomes and applications of the yoga practice* | Being present and cultivating awareness | 6 | |  |
|  |  | Consistency and persistence as key | 1 | |  |
|  |  | Application of practice to emotions, thoughts and behaviours | 23 | |  |
|  |  | Mental and spiritual benefits | 5 | |  |
|  |  | Steadiness, strength and focus | 2 | |  |
|  | *Adapting the practice and learning from challenges* | Adaptations due to COVID | 13 | |  |
|  |  | Navigating changes and adapting the practice | 35 | |  |
|  |  | Physically and mentally challenged by the practice | 21 | |  |
|  | *Expanding the yoga practice through additional elements* | Additional practice to balance or complement AY practice | 17 | |  |
|  |  | Study of the eight limbs of yoga | 14 | |  |
|  |  | Support from the teacher to adapt the practice and embrace changes | 4 | |  |

**Supplement 14.** *Example of participant’s response spanning across overarching theme “Yoga as an embodied framework to be used on and off the mat”*

| **Aspect of Yoga** | **Yoga** | **Yoga Philosophy** | **Yoga Practice** |
| --- | --- | --- | --- |
| **Theme** | *Yoga is a way of being, living and seeing the world* | *An applied framework for the yoga asana practice and daily life* | *Outcomes and applications of the yoga practice* |
| **Sub-theme** | A way of living and perceiving the world | A way of being, thinking and interacting in the world |  |
| **Participant’s Quotes (P096)** | Yoga asana, action through physical expression. Yoga as a personal philosophy is founded upon the 8 limbs, foundation for experiencing and understanding the experience of a conscious life. | Yoga philosophy, the 8 limbs. Yoga is a path to follow in life | Starting each day with engaging with my body helps me experience and appreciate my own awareness on many levels. Each day that awareness is different. Being able to appreciate my changes helps me to remain aware instead of reactive in my actions throughout my day. I work hard on my asana. Discovering that everyday leads me to a great degree of kindness to myself. |

**Supplement 15**. *Examples of responses spanning overarching theme “Yoga as an embodied path for self-knowledge”*

| **Aspect of Yoga** | **Yoga** | **Yoga Philosophy** | **Yoga Practice** |
| --- | --- | --- | --- |
| **Theme** | *Yoga is a way of knowing and developing the self* | *A collection of teachings for the knowledge and the evolution of the self* | *Adapting the practice and learning from challenges* |
| **Sub-theme** | A method for self-inquiry and self-discovery | A path for the evolution of the self |  |
| **Participant’s Quotes (P130)** | I am looking for a way to be kind to myself and leave daily stress behind while I also find it motivating to move towards a goal, ie. seeing my improvement in postures week by week | A chance to detach and feel like my self | Slowly using what time I have to improve my postures and gain strength and flexibility |

**Supplement 16.** *Examples of participant’s response spanning overarching theme “Yoga as a path for spiritual awakening and connection”*

| **Aspect of Yoga** | **Yoga** | **Yoga Philosophy** | **Yoga Practice** |
| --- | --- | --- | --- |
| **Theme** | Yoga is a journey towards the realisation of the Self | A spiritual journey towards the understanding of reality, human nature and beyond | Expanding the yoga practice through additional elements |
| **Sub-theme** | Experiencing or practising a state of union or oneness | Understanding the nature of the self and the spiritual journey to the Self | Study of the eight limbs of yoga |
| **Participant’s Quotes (P032)** | Acceptance of the ebb and flow of the world and to its impermanence. Cultivating the ability to not be caught up in the external world in order to go deeper into the self, the body and the mind to find inner peace. | Various techniques and teachings used to achieve a certain state of oneness | I practice Ashtanga 5-6 days a week, I meditate regularly and read yogic texts and commentaries of yogic texts, as well as the history behind it. |
| **Participant’s Quotes (P083)** | Freedom from the suffering caused by my mind | The journey of an experiential inquiry into what everything really is | Morning: up at 5am, mantra at 5:30am, full primary from 6 to 7:30am followed by 30 minutes of pranayama and/or meditation. Evening: reading texts/teachings from 6 to 7pm, meditation from 7 to 8pm, bedtime |
